# Real-world safety comparison between cenobamate and lacosamide: a pharmacovigilance study based on the FDA Adverse Event Reporting System

**DOI:** 10.3389/fneur.2025.1625612

**Published:** 2025-09-02

**Authors:** Shenglan Shang, Dongxin Chen, Zhirui Song, Chengrui Zhou, Qiuyue Chang, Lu Xiang, Mengchen Yu, Yan Zhao, Weiliang Li, Fan Zhou, Airong Yu

**Affiliations:** ^1^Department of Clinical Pharmacy, General Hospital of Central Theater Command, Wuhan, China; ^2^School of Medicine, Wuhan University of Science and Technology, Wuhan, Hubei Province, China; ^3^Department of Neurosurgery, General Hospital of Central Theater Command, Wuhan, China

**Keywords:** epilepsy, cenobamate, lacosamide, adverse events, pharmacovigilance

## Abstract

**Background:**

Cenobamate (CNB) and lacosamide (LCM) are two common used third-generation anti-seizure medications (ASMs) for third-line treatment of the drug-resistant epilepsy. The real-world data on adverse events (AEs) related to them remains limited.

**Methods:**

All data obtained from the US Food and Drug Administration Adverse Event Reporting System (FAERS) database, covering the period from 2008 to 2024. The reporting odds ratio, proportional reporting ratio and bayesian confidence propagation neural network to assess and compare the safety signals of CNB and LCM for comparison.

**Results:**

A total of 50,323,324 AE reports were recorded, with 3,584 for CNB and 13,874 for LCM. The most significant signals were primarily in nervous system and psychiatric disorders, resembling those of traditional sodium channel blockers. Unreported AEs in the drug dispensatory were identified in LCM (multiple-drug resistance). Notable differences between LCM and CNB emerged: Certain numbers of AE signals associated with LCM were found in cardiac disorders, while no such relevant signals were detected for CNB; among the signals that detected in both drugs, most signals from CNB are stronger than those from LCM; The initial titration dose of CNB (12.5 mg, qd) reported a significantly higher number of AEs compared to the other dose groups.

**Conclusion:**

Choosing the right ASMs requires consideration of the type of epilepsy, the individual tolerance and potential severe toxicity of different medications. Although the disproportionality analysis is a hypothesis generating, we provide a reference for the clinical safety of CNB and LCM.

## 1 Introduction

Epilepsy is one of the most common and severe neurological disorders, affecting approximately 70 million people worldwide (1). Anti-seizure medications (ASMs) are the cornerstone of epilepsy treatment. Patients who fail to effectively control their seizures after trials with two properly dosed ASMs are classified as having drug-resistant epilepsy (DRE) (2). Focal seizures are the most common type of epileptic seizures and are more prone to drug resistance, with the drug resistance rate even exceeding 50% (3). Cenobamate (CNB) and lacosamide (LCM) are two commonly used third-generation ASMs for third-line treatment of focal DRE (4).

CNB, a novel oral ASM, was approved for monotherapy or adjunctive therapy in adults with focal seizures by the Food and Drug Administration (FDA) of United States in November 2019 and for adjunctive treatment of focal DRE by the European Medicines Agency (EMA) in March 2021 (5, 6). Studies also showed that CNB demonstrates remarkable superiority in the treatment of focal epilepsy (7, 8). CNB exerts its effect mainly through a dual mechanism. As a sodium channel blocker (SCB), it acts on voltage-gated sodium channels (VGSCs), suppressing persistent sodium currents instead of transient ones to diminish repetitive neuronal firing (9). Meanwhile, CNB serves as a positive allosteric modulator of γ-aminobutyric acid (GABA)-A receptors, augmenting neuronal inhibition (10). Adverse events (AEs) associated with CNB are generally mild to moderate, primarily involving nervous system symptoms such as somnolence, dizziness, diplopia, and gait or coordination issues (11). The incidence of treatment-emergent adverse events (TEAEs) may be related to faster titration rates and higher starting doses, with a higher incidence noted in patients using SCBs and benzodiazepines (e.g., clobazam) concurrently (12).

LCM, available in multiple formulations, received FDA and EMA approval in 2008 for the treatment of focal seizures, either as monotherapy or in combination (13). LCM is also the first third-generation ASM approved for sale in China. LCM is also a SCB, which selectively enhances the slow inactivation of VGSCs, thereby reducing pathological hyperexcitability without affecting the physiological activities of neurons (14). Additionally, it is reported that LCM interacts with collapsin response mediator protein-2 (CRMP-2), preventing abnormal neuronal connections linked to epilepsy (15). Common AEs associated with LCM include dizziness, headache, somnolence, diplopia, and arrhythmias, most of which are mild to moderate (16). Reducing the maintenance dose can alleviate or eliminate these adverse effects (17).

Patients with DRE typically require combination therapy with at least two ASMs, increasing the likelihood of drug interactions and adverse effects (18). However, real-world data on AEs related to newer-generation ASMs remains limited. The FDA Adverse Event Reporting System (FAERS), one of the largest pharmacovigilance databases globally, serves as a public, voluntary, spontaneous reporting system aimed at facilitating post-marketing safety monitoring (19). In this study, we extracted data from the FAERS database to identify safety signals, thoroughly assess, compare, and analyze real-world AEs related to CNB and LCM. We aim to enhance clinical awareness of the AEs associated with these two drugs and provide a reference for the clinical safety of novel ASMs.

## 2 Methods

### 2.1 Data source

All data for this study were obtained from the US Food and Drug Administration (FDA) Adverse Event Reporting System (FAERS) database, covering the period from Quarter 1 (Q1) of 2008 to Q3 of 2024. The raw data files were downloaded from the official FDA website.^1^ The FAERS database consists of seven primary data files: demographic and administrative information (DEMO), drug details (DRUG), reported adverse events (REAC), patient outcomes (OUTC), sources of reports (RPSR), drug therapy information (THER), and indications for drug use (INDI). The FAERS database follows international safety reporting guidelines from the International Conference on Harmonization, coding all AEs with preferred terms (PTs) from the Medical Dictionary of Regulatory Activities. PTs cover signs, symptoms, diagnoses, lab tests, and medical/family history. They can also be grouped into high-level group terms (HLGTs) and system organ classes (SOCs), or organized using Standardized MedDRA Queries (SMQs) for specific conditions.

### 2.2 Ethic approval

FARES database is a de-identified public database, thus this study not requiring any form of ethic approval.

### 2.3 Drug identification

Given the vast number of drug-related AE and drugs in the FAERS database, we focused our analysis on two specific drugs: the generic names “Cenobamate” and “Lacosamide,” along with their brand names “Xcopri,” “Motpoly XR” and “Vimpat.” This approach allowed us to effectively screen relevant reports for the targeted drugs.

### 2.4 Adverse event

In accordance with FDA guidelines for data deduplication, we selected the field labels PRIMARY_ID, CASE_ID, and FDA_DT from the DEMO table and sorted the data based on these labels. For reports that share the same CASE_ID, we retained only the report with the highest FDA_DT value. In instances where multiple reports have the same CASE_ID and FDA_DT, we kept only the report with the largest PRIMARY_ID value.

Two chief pharmacists categorized the AE reports based on standardized MedDRA queries (SMQs) and gathered clinical characteristics of patients, including gender, age, and AE outcomes. Importantly, this study places greater emphasis on AEs that are not documented in the descriptions of each drug or those that were previously undetected. With the assistance of these pharmacists and drug dispensatory, we were able to exclude drug indications from the AEs and identify AEs that were overlooked by healthcare professionals.

### 2.5 Statistical analysis

In this study, we employed the reporting odds ratio (ROR) and proportional reporting ratio (PRR) to assess the association between two groups of drugs. Higher values of ROR and PRR indicate a stronger relationship between the target drug and specific AEs. However, since relying on a single algorithm can introduce bias, we also utilized an alternative method known as the Bayesian Confidence Propagation Neural Network (BPNN) to further analyze the data and minimize false positive safety signals. A signal was identified if: (1) a ≥ 6, (2) ROR ≥ 2 with 95% CI > 1, (3) PRR > 2 with *χ*^2^ > 4, and (4) IC-2SD > 0. All disproportionality analyses in databases were followed READUS-PV guidelines (20). The ratio imbalance measurement algorithm was in Supplementary Table S1. Disproportionate measurement principles and signal detection standards were in Supplementary Table S2. Reports that did not meet these criteria were not considered signals and were excluded from this study.

After acquiring the enrollment data, we systematically compared the safety signals of PTs and SOCs for CNB and LCM. All analyses were conducted using Microsoft Excel 2019 or R (V4.1.2), while figures were created using python (v3.12).

## 3 Results

### 3.1 Baseline patient characteristics

**Figure 1:**
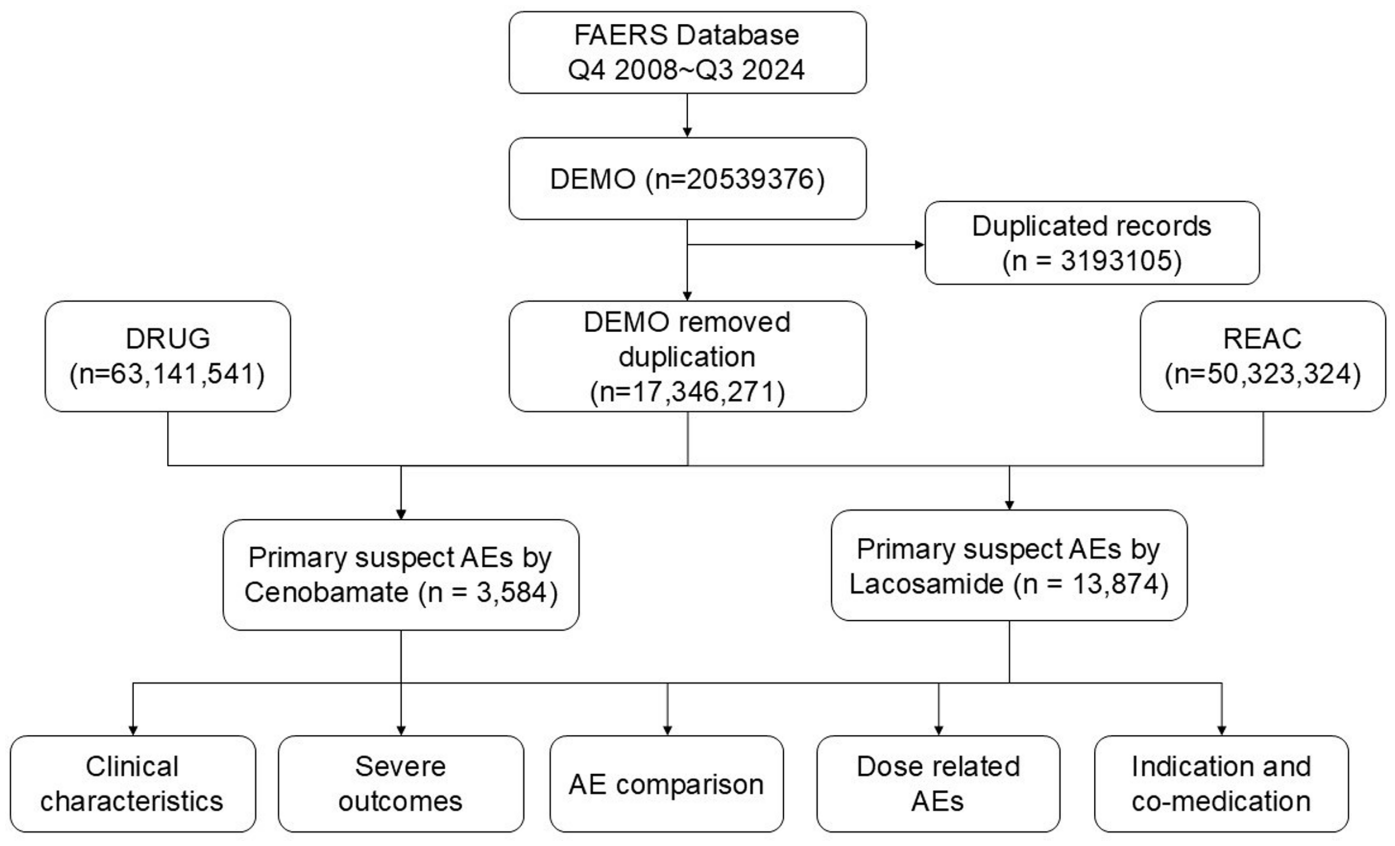
The flow chart of the study.

**Figure 2:**
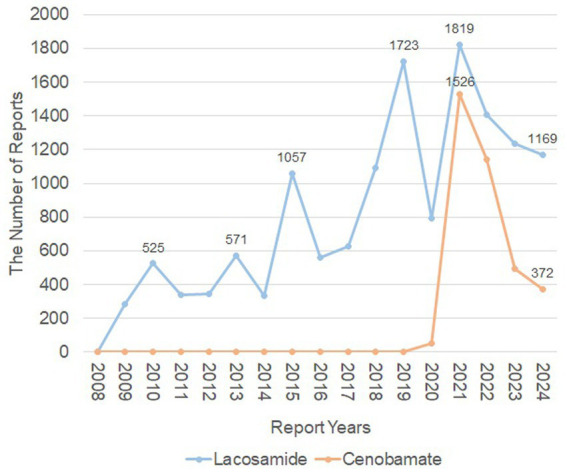
The number of adverse events reported yearly after the marketing of lacosamide and cenobamate. The orange line represented the reports of Cenobamate, while the blue line represented the reports of Lacosamide. X-axis shows the timeline when the drug was used, and Y-axis displays the number of reports per year.

The baseline characteristics of patients were presented in Table 1. From Q4 of 2008 to Q3 of 2024, a total of 50,323,324 AE reports were recorded from FAERS, in which 3,584 reports were associated with CNB, and 13,874 with LCM (Figure 1). This disparity is due to CNB’s later introduction, as it was added to the FAERS database in Q4 of 2020. The highest proportion of AE reports came from the United States, accounting for 58.2% of LCM-related reports and 89.6% of CNB-related reports. The number of AEs reported yearly after the marketing was shown in Figure 2.

**Table 1:** Characteristics of reports associated with lacosamide and cenobamate of the FAERS.

Index	Lacosamide (%)	Cenobamate (%)
Number of events	13,874	3,584
Gender		
Female	6,724 (48.5)	10 (0.3)
Male	5,491 (39.6)	13 (0.4)
Unknown	1,659 (12.0)	3,561 (99.4)
Age		
<18	1,019 (7.3)	1 (0.0)
18–49	3,016 (21.8)	14 (0.4)
50–79	3,133 (22.6)	4 (0.01)
≥80	585 (4.2)	0 (0)
Unknown	6,121 (44.1)	3,565 (99.5)
Serious outcomes		
Death	1,333 (9.6)	49 (1.4)
Disability	134 (1.0)	7 (0.2)
Life-threatening	367 (2.6)	47 (1.3)
Hospitalization	3,600 (25.9)	436 (12.2)
Other	4,866 (35.1)	391 (10.8)
Unknown	5 (0.0)	2 (0.1)
Reporter country		
United States	8,068 (58.2)	3,210 (89.6)
Japan	1,179 (8.5)	0 (0)
Germany	1,114 (8.0)	43 (1.2)
French/Britain	610 (4.4)	70 (2.0)
Other countries	2,903 (20.9)	261 (7.2)

CNB and LCM had the same top three indications, and the concomitant medications were also quite similar, including Levetiracetam, Lamotrigine and Topiramate (Table 2). Notably, CNB was frequently used in combination with LCM (3rd concomitant medication). The top 10 AEs were summarized in Table 3. The common AEs between CNB and LCM were in nervous system, such as somnolence (CNB *n* = 406, LCM *n* = 425), dizziness (CNB *n* = 344, LCM *n* = 694), balance disorder (CNB *n* = 140, LCM *n* = 213), memory impairment (CNB *n* = 120, LCM *n* = 289).

**Table 2:** Top 3 indications and top 5 concomitant medications and in AE reports of lacosamide and cenobamate.

Index	Lacosamide (*n*)	Cenobamate (*n*)
Indications	Epilepsy (3823)	Epilepsy (986)
	Seizure (3542)	Seizure (1069)
	Partial seizures (852)	Partial seizures (197)
Concomitant medication	Levetiracetam (2404)	Levetiracetam (144)
	Lamotrigine (894)	Lamotrigine (293)
	Carbamazepine (531)	Lacosamide (97)
	Topiramate (572)	Clobazam (245)
	Valproic acid (511)	Topiramate (113)

**Table 3:** Top 10 in the number of adverse event report of lacosamide and cenobamate.

Lacosamide	*n*	Cenobamate	*n*
Dizziness	694	Fatigue	432
Fall	584	Somnolence	406
Somnolence	425	Dizziness	344
Memory impairment	289	Fall	213
Bradycardia	220	Feeling abnormal	172
Balance disorder	213	Gait disturbance	142
Loss of consciousness	208	Balance disorder	140
Amnesia	196	Memory impairment	120
Multiple-drug resistance	177	Insomnia	95
Diplopia	174	Drug interaction	91

The number of AE reports of CNB and LCM in patients with various daily dose were presented in Table 4. The lowest dose of CNB (12.5 mg) was reported the highest number of AEs, while the 200 ~ 400 mg dose of LCM was reported the most AEs.

**Table 4:** Number of adverse event reports of lacosamide and cenobamate in patients over 18 years old with various daily dose.

Daily dose	Lacosamide (*n*)	Daily dose	Cenobamate (*n*)
≤100 mg	1209	12.5 mg	1952
100 mg ~ 200 mg	1540	25 mg	363
200 mg ~ 400 mg	1940	50 mg	377
>400 mg	521	100 mg	344
		150 mg	215

### 3.2 Disproportionality analyses

A total of 55 strong signals with an IC-2SD ≥ 1.0 were identified for CNB, while 98 were found for LCM, as shown in Table 5. First of all, nervous system disorders were the most prominent SOC in both medications, such as somnolence (CNB IC-2SD = 3.29, LCM IC-2SD = 1.55), balance disorder (CNB IC-2SD = 2.81, LCM IC-2SD = 1.65), dysarthria (CNB IC-2SD = 2.76, LCM IC-2SD = 1.01), eye movement disorder (CNB IC-2SD = 2.55, LCM IC-2SD = 1.12), sedation (CNB IC-2SD = 2.14, LCM IC-2SD = 1.24), ataxia (CNB IC-2SD = 1.94, LCM IC-2SD = 2.54), memory impairment (CNB IC-2SD = 1.88, LCM IC-2SD = 1.43) and aphasia (CNB IC-2SD = 1.85, LCM IC-2SD = 1.77). Other similar SOCs are psychiatric disorders (CNB 16 signals, IC-2SD range:1.02 ~ 2.99; LCM 21 signals, IC-2SD range: 1.00 ~ 2.89), general disorders and administration site conditions (CNB 9 signals, IC-2SD range:1.03 ~ 4.15; LCM 3 signals, IC-2SD range: 1.14 ~ 6.25), eye disorders (CNB 2 signals, IC-2SD range:1.71 ~ 4.12; LCM 1 signal: diplopia, IC-2SD = 3.8), injury, poisoning and procedural complications (CNB 1 signal: fall IC-2SD = 1.54, LCM 1 signal: fall IC-2SD = 1.27). CNB has 2 unique SOC classification of AE, such as social circumstances (2 signals, IC-2SD range: 1.20 ~ 1.46), respiratory, thoracic and mediastinal disorders (1 signal: hiccups, IC-2SD = 2.53). LCM has 6 unique SOC classification of AE, such as cardiac disorders (18 signals, IC-2SD range: 1.02 ~ 4.92), congenital, familial and genetic disorders (13 signals, IC-2SD range: 1.16 ~ 7.39), investigations (5 signals, IC-2SD range: 1.11 ~ 5.11), metabolism and nutrition disorders (3 signals, IC-2SD range: 1.04 ~ 1.84), pregnancy, puerperium and perinatal conditions (5 signals, IC-2SD range: 1.10 ~ 3.29), skin and subcutaneous tissue disorders (2 signals, IC-2SD range: 1.12 ~ 1.36), and vascular disorders (1 signal: systolic hypertension IC-2SD = 3.57). Additionally, signals with an IC-2SD ≥ 0 are also presented in Supplementary Table S3.

**Table 5:** Comparison of adverse event signals between lacosamide and cenobamate in various system organ classes.

SOCs/PTs	Lacosamide					Cenobamate				
	N	PRR	Chi_squared	ROR(CI025)	IC(IC-2SD)	N	PRR	Chi_squared	ROR(CI025)	IC(IC-2SD)
Cardiac disorders										
Atrial flutter	37	7.07	191.94	7.08 (5.12 ~ 9.78)	2.82 (2.35)	/	/	/	/	/
Atrioventricular block	93	19.2	1580.72	19.25 (15.68 ~ 23.63)	4.24 (3.94)	/	/	/	/	/
Atrioventricular block complete	100	24.23	2185.12	24.29 (19.92 ~ 29.61)	4.57 (4.28)	/	/	/	/	/
Atrioventricular block first degree	36	13.14	399.56	13.15 (9.47 ~ 18.26)	3.7 (3.22)	/	/	/	/	/
Atrioventricular block second degree	52	27.73	1310.93	27.77 (21.09 ~ 36.56)	4.76 (4.36)	/	/	/	/	/
Bradycardia	220	6.39	996.87	6.42 (5.62 ~ 7.34)	2.67 (2.48)	/	/	/	/	/
Bradycardia neonatal	14	17.55	215.5	17.56 (10.36 ~ 29.76)	4.11 (3.36)	/	/	/	/	/
Bundle branch block left	24	9.18	173.69	9.19 (6.15 ~ 13.73)	3.19 (2.61)	/	/	/	/	/
Bundle branch block right	18	6.42	81.93	6.42 (4.04 ~ 10.21)	2.68 (2.01)	/	/	/	/	/
Conduction disorder	11	10.71	96.04	10.71 (5.92 ~ 19.4)	3.41 (2.57)	/	/	/	/	/
Defect conduction intraventricular	6	26.64	144.95	26.64 (11.87 ~ 59.81)	4.71 (3.6)	/	/	/	/	/
Sinus arrest	23	24.97	518.88	24.98 (16.53 ~ 37.76)	4.61 (4.02)	/	/	/	/	/
Sinus bradycardia	48	7.94	289.48	7.95 (5.99 ~ 10.56)	2.98 (2.57)	/	/	/	/	/
Sinus node dysfunction	34	27.7	856.15	27.73 (19.73 ~ 38.95)	4.76 (4.27)	/	/	/	/	/
Supraventricular extrasystoles	8	3.97	17.73	3.97 (1.98 ~ 7.95)	1.99 (1.02)	/	/	/	/	/
Ventricular tachycardia	41	4.07	94.68	4.07 (3 ~ 5.54)	2.02 (1.58)	/	/	/	/	/
Electrocardiogram pr prolongation	21	48.6	942.38	48.63 (31.44 ~ 75.21)	5.55 (4.92)	/	/	/	/	/
Electrocardiogram qrs complex prolonged	17	5.5	62.29	5.5 (3.42 ~ 8.86)	2.45 (1.77)	/	/	/	/	/
Congenital, familial and genetic disorders										
Atrial septal defect	33	5.41	118.19	5.42 (3.85 ~ 7.62)	2.43 (1.93)	/	/	/	/	/
Brugada syndrome	9	13.81	105.77	13.81 (7.16 ~ 26.64)	3.77 (2.85)	/	/	/	/	/
Cardiac septal defect	10	20.28	180.4	20.29 (10.86 ~ 37.9)	4.32 (3.44)	/	/	/	/	/
Coarctation of the aorta	20	29.28	533.83	29.3 (18.8 ~ 45.65)	4.84 (4.2)	/	/	/	/	/
Congenital hydronephrosis	11	22.76	224.71	22.76 (12.54 ~ 41.33)	4.48 (3.64)	/	/	/	/	/
Cryptorchism	8	10.79	70.44	10.79 (5.38 ~ 21.64)	3.42 (2.45)	/	/	/	/	/
Cytogenetic abnormality	7	8.4	45.3	8.4 (3.99 ~ 17.66)	3.06 (2.04)	/	/	/	/	/
Fetal malformation	17	24.66	378.42	24.67 (15.26 ~ 39.87)	4.6 (3.91)	/	/	/	/	/
Hepatic arteriovenous malformation	7	545.77	2647.91	545.86 (224.55 ~ 1326.92)	8.57 (7.39)	/	/	/	/	/
Multiple congenital abnormalities	11	11.28	102.18	11.29 (6.23 ~ 20.44)	3.48 (2.65)	/	/	/	/	/
Polydactyly	7	8.84	48.32	8.84 (4.2 ~ 18.59)	3.13 (2.11)	/	/	/	/	/
Spina bifida	7	4.54	19.27	4.54 (2.16 ~ 9.54)	2.18 (1.16)	/	/	/	/	/
Trisomy 18	6	24.78	134.28	24.79 (11.05 ~ 55.62)	4.6 (3.5)	/	/	/	/	/
Eye Disorders										
Diplopia	174	10.46	1477.28	10.5 (9.04 ~ 12.2)	3.38 (3.16)	83	17.39	1277.97	17.51 (14.11 ~ 21.74)	4.12 (3.8)
Vision blurred	/	/	/	/	/	83	3.27	131.12	3.29 (2.65 ~ 4.08)	1.71 (1.39)
General disorders and administration site conditions										
Crying	/	/	/	/	/	22	3.16	32.45	3.16 (2.08 ~ 4.81)	1.66 (1.06)
Drug interaction	/	/	/	/	/	91	3.08	127.83	3.09 (2.52 ~ 3.8)	1.62 (1.32)
Drug intolerance	/	/	/	/	/	51	2.71	54.88	2.71 (2.06 ~ 3.57)	1.44 (1.03)
Fatigue	/	/	/	/	/	432	2.87	531.85	2.94 (2.67 ~ 3.24)	1.52 (1.38)
Feeling abnormal	/	/	/	/	/	172	3.65	331.89	3.69 (3.17 ~ 4.29)	1.87 (1.65)
Feeling drunk	27	5.5	98.94	5.5 (3.77 ~ 8.03)	2.45 (1.91)	35	24.98	801.33	25.06 (17.96 ~ 34.95)	4.63 (4.15)
Gait disturbance	/	/	/	/	/	142	3.69	279.36	3.73 (3.16 ~ 4.4)	1.88 (1.64)
Gait inability	/	/	/	/	/	32	5.25	110.02	5.26 (3.72 ~ 7.45)	2.39 (1.89)
Multiple-drug resistance	177	95.75	15413.47	96.17 (82.51 ~ 112.09)	6.48 (6.25)	/	/	/	/	/
Screaming	15	3.64	28.58	3.64 (2.19 ~ 6.04)	1.86 (1.14)	7	5.93	28.66	5.93 (2.83 ~ 12.45)	2.57 (1.55)
Injury, poisoning and procedural complications										
Fall	584	2.62	585.52	2.64 (2.43 ~ 2.87)	1.39 (1.27)	213	3.34	350.3	3.38 (2.95 ~ 3.87)	1.74 (1.54)
Investigations										
Anticoagulation drug level abnormal	8	72.84	535.59	72.86 (35.71 ~ 148.64)	6.11 (5.11)	/	/	/	/	/
Anticonvulsant drug level above therapeutic	9	23.59	191.05	23.59 (12.2 ~ 45.62)	4.53 (3.61)	/	/	/	/	/
Anticonvulsant drug level decreased	14	16.86	206.06	16.86 (9.95 ~ 28.58)	4.06 (3.31)	/	/	/	/	/
Anticonvulsant drug level increased	15	15.08	194.82	15.08 (9.06 ~ 25.1)	3.9 (3.17)	/	/	/	/	/
Blood sodium decreased	35	3.02	47.29	3.02 (2.17 ~ 4.22)	1.59 (1.11)	/	/	/	/	/
Metabolism and nutrition disorders										
Cell death	6	4.4	15.72	4.4 (1.98 ~ 9.82)	2.13 (1.04)	/	/	/	/	/
Hyperammonaemia	14	4.36	36.17	4.36 (2.58 ~ 7.38)	2.12 (1.37)	/	/	/	/	/
Marasmus	7	7.33	38.01	7.33 (3.49 ~ 15.4)	2.87 (1.84)	/	/	/	/	/
Nervous system disorders										
Eye movement disorder	16	3.53	28.99	3.53 (2.16 ~ 5.77)	1.82 (1.12)	13	10.05	105.67	10.06 (5.83 ~ 17.34)	3.33 (2.55)
Altered state of consciousness	58	4	130.11	4 (3.09 ~ 5.18)	2 (1.62)	/	/	/	/	/
Amnesia	196	4.63	557.19	4.65 (4.04 ~ 5.35)	2.21 (2)	41	3.38	68.88	3.39 (2.5 ~ 4.61)	1.76 (1.31)
Aphasia	86	4.24	212.49	4.25 (3.44 ~ 5.25)	2.08 (1.77)	30	5.17	100.81	5.18 (3.62 ~ 7.41)	2.37 (1.85)
Apraxia	6	6.34	26.84	6.34 (2.84 ~ 14.14)	2.66 (1.56)	/	/	/	/	/
Ataxia	62	7.53	349.07	7.54 (5.87 ~ 9.68)	2.91 (2.54)	15	6.35	67.51	6.36 (3.83 ~ 10.55)	2.66 (1.94)
Balance disorder	213	3.61	401.1	3.62 (3.17 ~ 4.15)	1.85 (1.65)	140	8.3	897.83	8.39 (7.1 ~ 9.91)	3.05 (2.81)
Brain fog	/	/	/	/	/	9	6.14	38.68	6.14 (3.19 ~ 11.82)	2.62 (1.7)
Cerebral disorder	18	3.71	35.48	3.71 (2.33 ~ 5.89)	1.89 (1.22)	/	/	/	/	/
Clumsiness	/	/	/	/	/	6	11.28	56.07	11.29 (5.06 ~ 25.15)	3.49 (2.4)
Cognitive disorder	97	3.08	136.07	3.09 (2.53 ~ 3.77)	1.62 (1.33)	/	/	/	/	/
Coordination abnormal	30	3.49	53.17	3.49 (2.44 ~ 5)	1.8 (1.28)	21	8.55	139.68	8.56 (5.58 ~ 13.14)	3.09 (2.48)
Dementia	52	2.9	64.56	2.9 (2.21 ~ 3.81)	1.53 (1.14)					
Disturbance in attention	96	2.65	98.32	2.65 (2.17 ~ 3.24)	1.4 (1.11)	47	4.53	129.41	4.55 (3.41 ~ 6.06)	2.18 (1.76)
Dizziness	/	/	/	/	/	344	3.67	674.26	3.76 (3.37 ~ 4.18)	1.88 (1.72)
Drop attacks	14	35.5	456.37	35.51 (20.87 ~ 60.41)	5.11 (4.35)	/	/	/	/	/
Drug withdrawal convulsions	23	18.81	382.2	18.82 (12.47 ~ 28.42)	4.21 (3.62)	/	/	/	/	/
Dysarthria	63	2.59	61.34	2.59 (2.02 ~ 3.32)	1.37 (1.01)	61	8.77	419.62	8.82 (6.85 ~ 11.34)	3.13 (2.76)
Dysgraphia	/	/	/	/	/	8	6.12	34.24	6.13 (3.06 ~ 12.26)	2.61 (1.65)
Dyslexia	6	7.88	35.81	7.88 (3.53 ~ 17.58)	2.97 (1.88)	/	/	/	/	/
Dysstasia	/	/	/	/	/	29	5.08	94.87	5.09 (3.53 ~ 7.32)	2.34 (1.81)
Febrile convulsion	8	10.86	70.99	10.86 (5.41 ~ 21.79)	3.43 (2.46)	/	/	/	/	/
Hypersomnia	/	/	/	/	/	88	16.2	1250.72	16.31 (13.22 ~ 20.13)	4.01 (3.71)
Lethargy	/	/	/	/	/	58	5.44	210.21	5.46 (4.22 ~ 7.07)	2.44 (2.07)
Loss of consciousness	208	2.57	199.29	2.58 (2.25 ~ 2.95)	1.36 (1.16)	/	/	/	/	/
Memory impairment	289	3.04	395.77	3.06 (2.72 ~ 3.43)	1.6 (1.43)	120	4.41	317.26	4.45 (3.72 ~ 5.33)	2.14 (1.88)
Motor dysfunction	/	/	/	/	/	8	4.19	19.4	4.19 (2.09 ~ 8.38)	2.07 (1.1)
Nystagmus	27	8.05	165.6	8.05 (5.52 ~ 11.76)	3 (2.45)	7	7.27	37.79	7.27 (3.46 ~ 15.27)	2.86 (1.84)
Sedation	50	3.13	72.34	3.13 (2.37 ~ 4.14)	1.64 (1.24)	29	6.35	130.56	6.36 (4.42 ~ 9.16)	2.66 (2.14)
Slow speech	/	/	/	/	/	11	23.41	234.74	23.43 (12.95 ~ 42.39)	4.54 (3.71)
Somnolence	425	3.24	657.37	3.26 (2.96 ~ 3.59)	1.69 (1.55)	406	10.82	3622.68	11.18 (10.13 ~ 12.35)	3.43 (3.29)
Speech disorder	99	2.85	118.66	2.85 (2.34 ~ 3.48)	1.51 (1.22)	36	3.62	68.33	3.63 (2.62 ~ 5.04)	1.86 (1.38)
Syncope	157	2.38	125.98	2.39 (2.04 ~ 2.79)	1.25 (1.02)	/	/	/	/	/
Tongue biting	7	5.52	25.79	5.52 (2.63 ~ 11.6)	2.46 (1.44)	/	/	/	/	/
Tremor	/	/	/	/	/	79	2.53	73.12	2.54(2.03 ~ 3.17)	1.34(1.01)
Bradyphrenia	/	/	/	/	/	6	4.52	16.44	4.52(2.03 ~ 10.08)	2.18(1.08)
Pregnancy, puerperium and perinatal conditions										
Abortion spontaneous	131	4.94	410.24	4.95 (4.17 ~ 5.88)	2.3 (2.05)	/	/	/	/	/
Hydrops foetalis	8	19.49	138.19	19.5 (9.7 ~ 39.2)	4.26 (3.29)	/	/	/	/	/
Premature baby	58	2.78	66.14	2.79 (2.15 ~ 3.6)	1.47 (1.1)	/	/	/	/	/
Premature delivery	49	4.22	120.14	4.23 (3.19 ~ 5.6)	2.07 (1.67)	/	/	/	/	/
Stillbirth	19	5.78	74.71	5.78 (3.68 ~ 9.07)	2.52 (1.88)	/	/	/	/	/
Psychiatric disorders										
Mental impairment	/	/	/	/	/	18	3.86	38.2	3.87 (2.44 ~ 6.14)	1.95 (1.29)
Abnormal behavior	107	4.17	256.66	4.17 (3.45 ~ 5.05)	2.05 (1.78)	39	5.3	136.11	5.32 (3.88 ~ 7.28)	2.41 (1.95)
Acute psychosis	15	9.38	111.46	9.38 (5.65 ~ 15.59)	3.22 (2.5)	/	/	/	/	/
Affective disorder	23	4.42	60.77	4.43 (2.94 ~ 6.67)	2.14 (1.55)	/	/	/	/	/
Aggression	152	4.72	444.57	4.74 (4.04 ~ 5.56)	2.24 (2)	31	3.36	51.42	3.37 (2.37 ~ 4.79)	1.75 (1.24)
Agitation	127	2.68	133.85	2.69 (2.26 ~ 3.2)	1.42 (1.17)	/	/	/	/	/
Anger	89	3.91	192.66	3.92 (3.18 ~ 4.83)	1.97 (1.66)	34	5.23	116.1	5.24 (3.74 ~ 7.34)	2.38 (1.9)
Apathy	/	/	/	/	/	14	5.14	46.64	5.14 (3.05 ~ 8.69)	2.36 (1.61)
Behavior disorder	34	10.48	289	10.48 (7.48 ~ 14.69)	3.38 (2.89)	11	11.8	108.42	11.81 (6.53 ~ 21.34)	3.56 (2.72)
Communication disorder	/	/	/	/	/	6	8.57	40.07	8.58 (3.85 ~ 19.11)	3.1 (2.01)
Confusional state	/	/	/	/	/	77	2.55	72.95	2.56 (2.05 ~ 3.21)	1.35 (1.02)
Delirium	67	3.05	91.91	3.05 (2.4 ~ 3.88)	1.6 (1.25)	/	/	/	/	/
Dysphemia	11	3.77	22.37	3.78 (2.09 ~ 6.82)	1.91 (1.08)	11	13.22	123.84	13.23 (7.32 ~ 23.91)	3.72 (2.89)
Emotional disorder	/	/	/	/	/	20	3.36	33.22	3.37 (2.17 ~ 5.22)	1.75 (1.12)
Homicidal ideation	12	4.76	35.47	4.76 (2.7 ~ 8.39)	2.25 (1.44)	/	/	/	/	/
Impulse-control disorder	6	4.5	16.28	4.5 (2.02 ~ 10.03)	2.17 (1.07)	/	/	/	/	/
Inappropriate affect	6	5.58	22.46	5.58 (2.5 ~ 12.45)	2.48 (1.38)	/	/	/	/	/
Irritability	126	3.14	182.93	3.14 (2.64 ~ 3.74)	1.65 (1.39)	36	3.13	52.2	3.14 (2.26 ~ 4.35)	1.65 (1.17)
Logorrhoea	10	4.3	25.27	4.3 (2.31 ~ 8.01)	2.1 (1.23)	/	/	/	/	/
Mood altered	/	/	/	/	/	25	4.85	76.25	4.85 (3.28 ~ 7.19)	2.28 (1.71)
Mood swings	/	/	/	/	/	21	3.42	36	3.43 (2.23 ~ 5.26)	1.77 (1.16)
Paranoia	34	3.09	48.05	3.09 (2.21 ~ 4.33)	1.63 (1.14)	/	/	/	/	/
Persecutory delusion	10	6.45	45.85	6.45 (3.47 ~ 12.02)	2.68 (1.81)	/	/	/	/	/
Personality change	26	4.19	62.95	4.19 (2.85 ~ 6.16)	2.06 (1.51)	13	7.32	70.86	7.33 (4.25 ~ 12.63)	2.87 (2.1)
Psychomotor retardation	11	6.15	47.24	6.15 (3.4 ~ 11.13)	2.62 (1.78)	/	/	/	/	/
Psychotic disorder	71	3.81	146.65	3.81 (3.02 ~ 4.81)	1.93 (1.59)	/	/	/	/	/
Staring	14	9.27	102.53	9.27 (5.48 ~ 15.69)	3.2 (2.45)	7	16.17	99.23	16.18 (7.7 ~ 33.98)	4.01 (2.99)
Suicidal ideation	141	2.36	110.72	2.37 (2.01 ~ 2.79)	1.24 (1)	54	3.16	79.96	3.17 (2.43 ~ 4.15)	1.66 (1.27)
Respiratory, thoracic and mediastinal disorders										
Hiccups	/	/	/	/	/	14	9.71	109.12	9.72 (5.75 ~ 16.42)	3.28 (2.53)
Skin and subcutaneous tissue disorders										
Drug eruption	34	3.04	46.58	3.05 (2.18 ~ 4.27)	1.6 (1.12)	/	/	/	/	/
Lichenoid keratosis	7	5.22	23.8	5.22 (2.49 ~ 10.97)	2.38 (1.36)	/	/	/	/	/
Social circumstances										
Impaired quality of life	/	/	/	/	/	11	4.09	25.67	4.09 (2.27 ~ 7.4)	2.03 (1.2)
Impaired work ability	/	/	/	/	/	18	4.35	46.42	4.36(2.74 ~ 6.92)	2.12(1.46)
Vascular disorders										
Systolic hypertension	7	24.67	155.87	24.67(11.68 ~ 52.13)	4.6(3.57)	/	/	/	/	/

PTs, Preferred Terms; SOCs, System Organ Classes; N is the number of reported adverse events; IC (IC-2SD), information component (lower end of the 95% confidence interval); /, IC-2SD value of the adverse event is less than 1.0.

### 3.3 Comparison of safety signals between LCM and CNB

**Figure 3:**
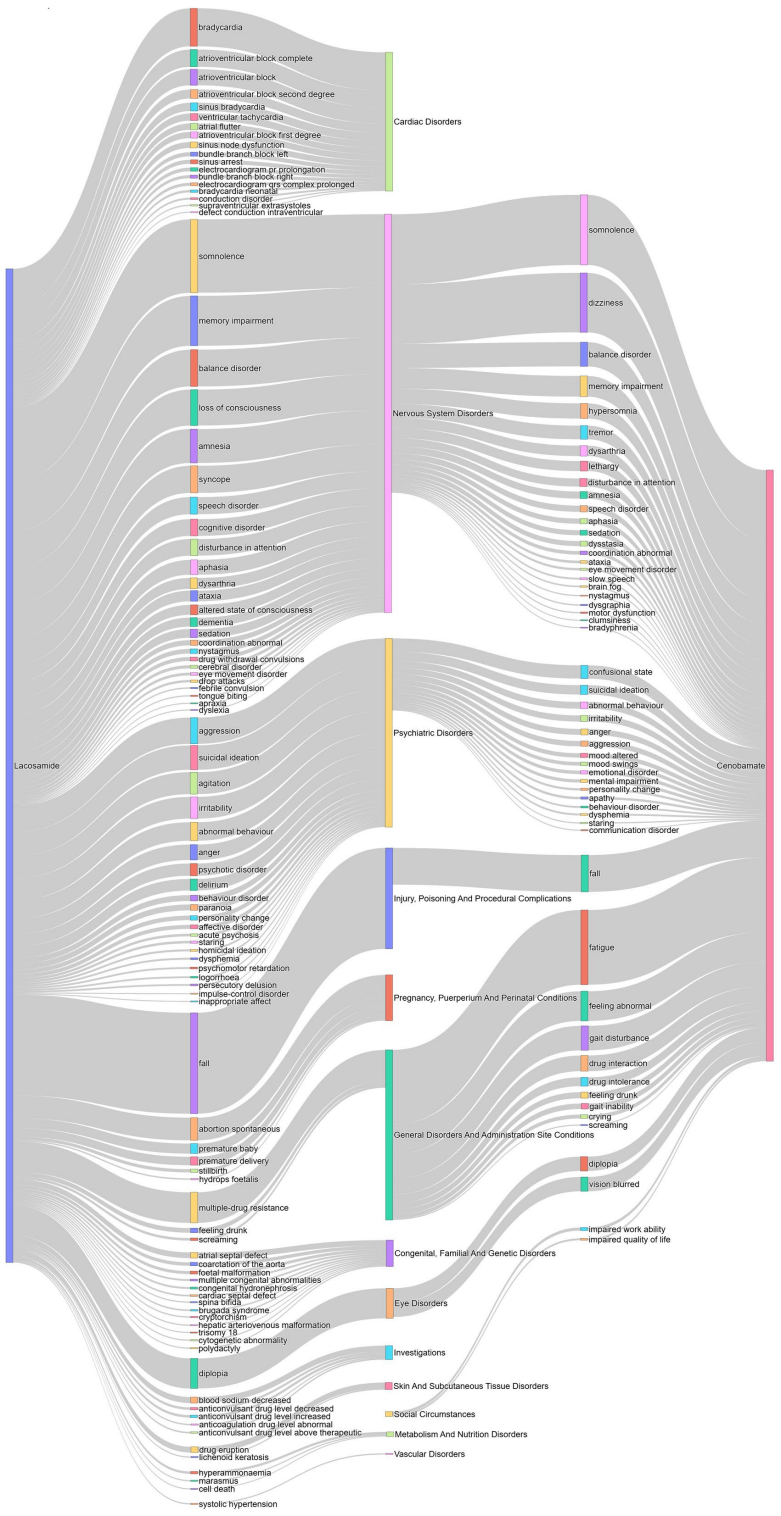
Comparison of adverse event signals between lacosamide and cenobamate. The lines on both ends correspond to the drug and its adverse events signals or the signals and its systems organ classes. The length of each bar indicates the number of the corresponding signals. The signals for Lacosamide emerge from the left, while those for Cenobamate originate from the right. The different signals converge in the middle bar, which represents the corresponding system organ classes.

Sankey diagram was used to compare safety signals between CNB and LCM (Figure 3). The length of each bar reflects the frequency of the respective signals. Different signals converge in the central bar, representing the corresponding SOCs. Compared with LCM, CNB’s AE had fewer types of SOCs and more concentrated on nervous system, psychiatric disorders, general disorders and administration site condition, and eye disorders.

**Figure 4:**
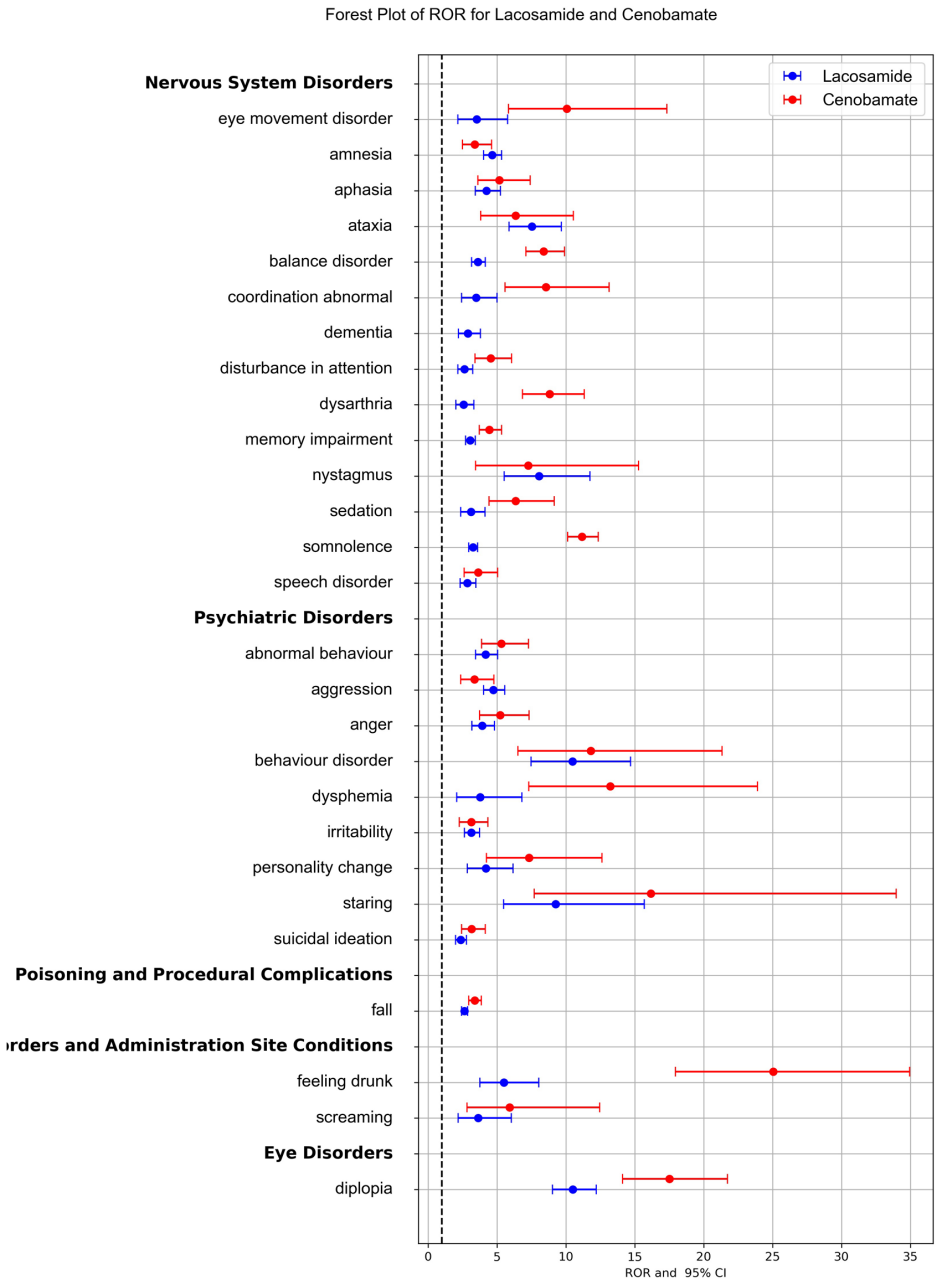
Reporting Odds Ratios (RORs) comparison for adverse events associated with lacosamide and cenobamate. The blue line represented the reports of Lacosamide, and the red line represented the reports of Cenobamate. Only adverse events signal appearing in both drugs were presented here for RORs comparison.

The forest plot of the ROR for LCM and CNB compared the signal strength of the two drugs, highlighting significant differences in the risk of specific AEs. Despite its shorter time on the market and fewer total AE reports, CNB exhibited stronger signal strength for most AEs (Figure 4).

## 4 Discussion

Based on the FAERS database from 2008 to 2024, we comprehensively compared the AE risks of CNB and LCM. The same AE of these two drugs were similar to traditional SCB reactions, such as dizziness, ataxia, feeling drunk, balance disorder, and diplopia (21). Unreported AEs in the drug dispensatory were identified, such as high signals for multiple-drug resistance in LCM. Notably, AEs between the two drugs showed differences: (1) Certain numbers of AE signals associated with LCM were found in cardiac disorders, reproductive toxicity, and genetic disorders, et al., while no such relevant signals were detected for CNB; (2) Among the signals that detected in both drugs, most signals from CNB are stronger than those from LCM, such as eye movement disorder, coordination abnormal, dysarthria, somnolence, feeling drunk, dysphemia, diplopia, et al.; (3) The initial titration dose of CNB (12.5 mg, qd) reported a significantly higher number of AEs compared to the other dose groups.

### 4.1 Nervous system disorders and psychiatric disorders

Nervous system disorders and psychiatric disorders are the most common AEs of ASMs, sometimes reaching up to 20%, and are linked to poor patient compliance and treatment discontinuation (22). Some AEs associated with CNB were the same as the traditional SCBs, such as dizziness, diplopia, and gait disturbance, and the incidence is even higher when it is used in combination with SCBs (23). It is worth noting that the half-maximal inhibitory concentration of the persistent sodium current is approximately 53 mM, while the half-maximal effective concentration of the GABA-A receptor is between 42 and 194 mM. Due to the dual mechanisms of CNB, as the dose of CNB increases, more GABA-A receptors will be involved, which will not only produce therapeutic effects but also cause potential AEs in the nervous system (11). Central nervous system-related AEs are frequently reported for CNB, including somnolence, dizziness, headache, and fatigue, with an incidence of 5.4%, primarily mild to moderate, and more frequent in patients on multiple ASMs (12, 24). These AEs typically appear during titration (usually in the first days/week) and can resolve spontaneously, but dosage reduction of concomitant may be required (12, 24). Our analysis also found that initial titration dose of CNB were reported more AEs than other dose. Dizziness and somnolence are significant concerns for patients and major reasons for discontinuation with an incidence of about 22%, which might due to its effect on GABA-A receptors. In our study, the signal for somnolence and dizziness with CNB was also notably higher than with LCM. Clinical data indicate that slower titration can reduce the incidence of somnolence, dizziness, and fatigue in patients taking CNB (25). Experts recommend evening dosing or reducing the dosage of co-administered ASMs to mitigate AEs, especially when combined with benzodiazepines or SCBs, to lower the risk of synergistic adverse effects (26).

LCM also causes AEs similar to traditional SCBs, such as dizziness, headache, fatigue, diplopia, ataxia, and balance disorder. The most frequent TEAEs leading to discontinuation are dizziness and ataxia. Dizziness is a common AE associated with LCM, with an incidence of 8.3–55%, mostly mild (16). The incidence of dizziness and discontinuation increases with dosage, with severe dizziness reported in higher-dose groups (600 mg/day) (17). Dizziness is most likely to occur in the first 3 months of LCM use, with a rate during titration 3 ~ 4 times higher than during maintenance (27). Somnolence occurs less frequently than dizziness and headache, with an incidence of about 1.6 ~ 15% (28). Recent studies on children with epilepsy under 4 years old suggest that somnolence is a common AE (29). Most somnolence AEs occur during titration, with no clear correlation to dosage (30).

Patients with refractory epilepsy are more likely to experience psychiatric disorder (31). In our study, there was a notable signal for LCM associated acute psychosis. It was reported that the incidence of psychiatric AEs in patients treated with LCM ranges from 0.3 to 3.1%, often leading to discontinuation or serious consequences (16, 31). Study showed no significant relationship between LCM dosage and psychiatric disorder (32). Psychiatric disorder typically occur within hours to 2 weeks after administration, with a higher incidence in patients with pre-existing mental disorders (33). Large genomic studies indicate that various voltage-gated sodium channels are involved in the pathogenesis of psychosis, and over activity of CRMP-2 is also linked to psychiatric symptoms (34). LCM may enhance the slow inactivation of voltage-gated sodium channels and modulate CRMP-2, potentially leading to negative psychiatric AEs in patients.

### 4.2 Cardiac and vascular disorders

Increasing evidence indicates that certain ASMs, particularly SCBs, are linked to a higher risk of cardiac disorder (35). ASMs prolongs the QT interval, either by closing ion channels or delaying their opening to affect heart rhythm, which is a pathophysiological basis for ASM induced arrhythmias. Specifically, SCBs act on VGSCs, which is crucial ion channels responsible for generating action potentials, playing a significant role in neuronal excitability and epilepsy. Besides nervous system, VGSCs are also expressed in cardiac tissue and crucial for maintaining heart rhythm (36). Disruption of these channels can lead to arrhythmias and conduction blocks.

LCM’s inhibition of sodium channels may delay cardiac conduction below the atrioventricular bundle level, triggering arrhythmias. Although cardiac sodium channels are less sensitive to LCM blockade than neuronal ones, in vitro experiments confirm that LCM affect both neuronal and cardiac activities (37). Research confirms LCM affects cardiac sodium channels (hNav1.5) and is associated with electrocardiograph (ECG) changes, atrial fibrillation, and AV block (38). It can inhibit conduction in the His-Purkinje system, resulting in QRS prolongation and potentially causing recurrent arrhythmias and ventricular tachycardia (39). Consequently, the FDA contraindicates LCM for patients with existing second-degree or complete AV block and warns of increased atrial arrhythmias in those with diabetes or cardiovascular disease (40). Reports of sinus bradycardia and AV block have surfaced since LCM’s introduction, with severe cases leading to atrial fibrillation, QRS prolongation and cardiac arrest (41). This is largely consistent with our research findings, where strong signal were found in LCM associated cardiac AEs, such as various degrees of atrioventricular block, electrocardiogram PR prolongation, electrocardiogram QRS complex prolongation, sinus arrest, and bradycardia.

The correlation between cardiac AEs and LCM dose remains controversial. Some studies suggest that cardiac AEs are dose dependent, especially under loading doses (42–45). However, other research indicates that low dose LCM also led to cardiac AEs (42, 46). Moreover, most cardiac AEs related to LCM occur during the titration period, and their incidence shows a downward trend over time (47). Our study found no clear relationship between the reported number of AEs and the LCM dose. The reported number of AEs showed a slight upward trend with the increase in LCM dose, but dropped sharply in the group with a dosage > 400 mg. This could be attributed to the inherent limitations of the database, given that the proportion of unreported dosages is substantial.

Several large-scale studies have found that the overall incidence of cardiac adverse events (AEs) related to oral Lacosamide (LCM) ranges from 0.7 to 1.2%, with mild symptoms. Most of them do not require intervention or can be relieved after drug discontinuation (48, 49). There are certain differences between the AEs of intravenous and oral administrations. A study in South Korea showed that the incidence of cardiac AEs was relatively high (32.9%) when LCM was rapidly administered intravenously (400 mg within 10 to 20 min), especially the first-degree atrioventricular block (22.4%), which led to a prolonged average PR interval (50). However, some studies suggest that although intravenous LCM may have certain impacts on electrocardiogram parameters and blood pressure, the changes are mild and clinically insignificant, and there’s no need to stop LCM or implant a pacemaker. Considering the risk of seizures, slow intravenous administration of LCM seems to be a relatively safe option. When the infusion time of LCM is slowed down to 30 min, the cardiac side effects are low and it does not affect the effectiveness of seizure control (51). But for emergency situations like status epilepticus, a faster infusion rate may be required, and the safety of LCM in such cases needs further research. In our study, the AE signals of bradycardia and ventricular tachycardia were relatively high. Studies have shown that ventricular tachycardia is common among the arrhythmias caused by LCM (45). ST segment elevation has been reported after using LCM (52). Thus, clinically, prior to LCM administration, it’s crucial to ascertain patients’ heart disease history. For those with arrhythmia predisposing factors like cardiac conduction disease history, concurrent use of cardiac conduction affecting drugs, or diabetic neuropathy, LCM should be used cautiously. After LCM is administered, attention should be paid to monitoring patients’ cardiac function and electrocardiogram changes, especially in the first month after starting treatment. If necessary, the dosage should be reduced or the drug should be discontinued. In addition, when other SCBs like carbamazepine and phenytoin are used simultaneously with LCM, they may increase cardiac AEs, so caution should be exercised when combining them.

### 4.3 Pregnancy, puerperium and perinatal conditions

The incidence of congenital malformations in pregnant women with epilepsy not taking ASMs is similar to that of the general population, around 2 ~ 4% (53). During pregnancy, especially in the first trimester, the incidence of congenital malformations associated with ASM use is 2 ~ 3 times higher, approximately 4 ~ 8% (53, 54). An observational study of pregnant women exposed to ASMs indicated that LCM is the most commonly used third-generation ASM (13). Most patients were exposed to LCM early in pregnancy, which is associated with an increased risk of malformations, as this period is critical for embryonic organ development. The overall incidence of congenital malformations with LCM combination therapy is higher than with monotherapy, consistent with reports on other ASMs (53, 54). A case report showed that three pregnant women exposed to LCM, showing good efficacy and safety during pregnancy and breastfeeding, with no teratogenic or potential toxic effects (53). However, high concentrations of LCM can pharmacologically affect the placenta, impacting fetal folate supply, so plasma levels must be closely monitored during pregnancy to adjust the dosage dynamically (55). Our study identified reproduction toxicity high-signal related to LCM, including fetal malformation, hydrops foetalis, bradycardia neonatal, premature baby, and abortion spontaneous, consistent with existing literature. This finding was drawn without restricting the analysis to either LCM monotherapy or its combination with other drugs. There is currently no clear data on whether CNB is teratogenic. For pregnant patients, it is recommended to use ASMs with relatively high safety, such as lamotrigine, levetiracetam, or oxcarbazepine (56). Additionally, pregnant women should avoid combining multiple ASMs, especially high-risk teratogenic medications like valproic acid. A careful balance of risks and benefits should be considered in the context of effective seizure control and pregnancy toxicity when determining the treatment plan.

### 4.4 Drug interaction with other ASMs

Although monotherapy is the first choice for epilepsy treatment, some patients may require two or more ASMs. For patients with focal epilepsy, combination therapy is more effective after the first anti-epileptic treatment fails. As LCM and CNB are often used in DRE patients, who typically use multiple ASMs, increasing the number of combined ASMs heighten the risk of AEs due to pharmacokinetic and pharmacodynamic interactions. In such cases, a common approach is to adjust the doses of existing ASMs rather than discontinue the newly added ASM. This can improve patient tolerance and safety while titrating the new ASM to an effective dose. Using ASMs with similar mechanisms lead to excessive pharmacodynamic effects and AEs, particularly during later titration phases when high doses of similar mechanism ASMs are employed, such as SCBs and benzodiazepines (e.g., clobazam) (57). These interactions occur at the ASM target sites, altering pharmacological effects without changing plasma concentrations. LCM and CNB both carry a higher risk of AEs when used with SCBs (1). Our AE signal analysis found that the low-dose CNB group (12.5 mg, qd) reported more AEs, likely due to a higher incidence of AEs during early titration (1^st^ week) (12, 24). In contrast, the 200 mg ~ 400 mg dose range of LCM reported the most numbers of AEs, possibly because this range is the most commonly used maintenance dose with the largest population of patients (40).

While LCM and CNB do not have significant clinical pharmacokinetic interactions, the incidence of dose-related nervous system AEs (such as dizziness, somnolence, and ataxia) increases with combination therapy, likely due to pharmacodynamic interactions, as both act on voltage-gated sodium channels, albeit differently (9). If the combined LCM dose is high (≥500 mg/day), these interactions may occur relatively early in CNB titration. Therefore, it is recommended to actively reduce the LCM dose early in CNB titration (e.g., decrease by 25% every 2 weeks as needed) to mitigate potential adverse effects from pharmacodynamic interactions (57). If patients are already on two or more SCBs, it is advisable to proactively lower the SCB dose or discontinue them when adding CNB (18).

It is worth noting that clobazam is a long-acting benzodiazepine. It can bind to GABA-A receptors to enhance GABAergic neurotransmission. When combining used with CNB, it has an additive effect on the action of GABA, increasing the inhibitory effect on neurons and resulting in significant somnolence. In addition, CNB significantly increase the concentration of clobazam and/or its active metabolite (N-desmethylclobazam) by 2–6 times via inhibiting CYP2C19. Due to the pharmacokinetic and pharmacodynamic interactions between the two drugs, their combination may lead to dual interactions and thus cause serious adverse events (SAEs) (58, 59). Our results also indicate that clobazam is a commonly used drug in combination with CNB (ranking fourth), so caution should be exercised to avoid or carefully manage the use of benzodiazepines and their derivatives (40). Studies have found that after the initiation of CNB treatment, among the different classes of concomitant ASMs, clobazam shows the greatest reduction in drug load (60, 61). When CNB is used in combination with clobazam, reducing the dose of clobazam as early as possible helps CNB achieve the optimal titration dose (57, 62). When CNB is combined with a high dose of clobazam (≥40 mg/d), reducing the latter to a low dose of 5-10 mg is beneficial for reducing AEs and controlling epileptic seizures (18, 58, 63).

### 4.5 Other AEs signals

Our study also identified a strong signal for multiple drug-resistant AEs related to LCM, which is not mentioned in its FDA labeling. The mechanisms of ASM resistance are unclear, with past studies suggesting hypotheses like target alteration, transport proteins, and pharmacokinetic changes (64). LCM’s anticonvulsant effect primarily involves binding to and inactivating sodium channel subunits. If the expression or structure of these subunits is altered, LCM may lose its efficacy, leading to drug-resistant epilepsy (65). Recent studies on pediatric epilepsy patients have shown an increasing rate of LCM resistance (65). Patients with a long standing illness or those unresponsive to initial ASM treatment, irrespective of prior drugs, are at an elevated risk of developing drug resistance. Moreover, a protracted disease course correlates with an increased ASM resistance risk (66). Furthermore, choosing an inappropriate ASM monotherapy early in the disease course may severely affect the sensitivity to later drugs. Resistance is highly specific to the type of ASM (67). Animal studies have shown that repeated early administration of sodium - channel - blocking ASMs promote drug resistant chronic seizures (68). Nevertheless, we need to note that since LCM is a third-line treatment for focal DRE, it is not used as a first- or second-line option. This finding of multiple drug-resistant AEs may not be exclusive to LCM; it could also reflect the practice related to associated ASMs that patients used previously. It is also commonly observed that ASMs reduce seizures but may increase AEs (69).

Our results indicated high signals for diplopia with both LCM and CNB, which is often associated with SCBs (70). If diplopia persists for ≥ 3 days, it’s recommended to reduce the dosage of SCBs. In this study, CNB was associated with AEs related to eye disorders, such as vision blurred (*n* = 83, IC-2SD = 1.39) and diplopia (*n* = 83, IC-2SD = 3.8), while LCM primarily showed diplopia with slightly lower signal strength (*n* = 174, IC-2SD = 3.16). Studies have demonstrated that diplopia is a common ophthalmic AEs (16), which is consistent with our findings.

## 5 Limitation

Several limitations of our study should be noted. First, the FAERS database, being a spontaneous reporting system, may contain duplicate reports and inconsistent symptom descriptions, leading to inaccuracies in AE incidence calculations. Missing information on complications, dosage, and medical history in AE reports limits the ability to assess safety comprehensively. Second, AE reports primarily come from the U. S. and Europe, which may not represent all populations due to ethnic differences. Third, methods like ROR, PRR and BPNN indicate statistical associations, not causality, necessitating further clinical studies for validation. Fourth, epilepsy patients often use multiple medications, increasing the risk of interactions. This study focused on monotherapy and did not account for combinations or specific dosages, suggesting future research should incorporate combined drug signal detection. Fifth, varying market entry times for LCM and CNB may lead to reporting bias, complicating direct safety comparisons. Larger future trials may uncover more adverse signals for CNB. Lastly, since this study does not specifically use a registry for pregnant women, assumptions about adverse effects related to these drugs in pregnancy is limited.

## 6 Conclusion

Early AEs affect compliance, diminish quality of life, and delay the achievement of optimal therapeutic doses. Research indicates that AEs associated with ASMs remain a leading cause of treatment failure and reduced quality of life in epilepsy patients (71). Achieving successful epilepsy management hinges on balancing effective seizure control with minimizing AEs. Choosing the right ASM requires consideration of the type of epilepsy, as well as the individual tolerance and potential severe toxicity of different medications in patients. By thoroughly analyzing and comparing the AEs of LCM and CNB, we provide valuable insights for assessing the clinical safety of the two medications. Our study emphasizes the importance of vigilant monitoring of patients undergoing treatment and contributes to optimizing the therapeutic use of ASMs in clinical practice.

## Data Availability

Publicly available datasets were analyzed in this study. This data can be found at: www.FDA.gov.

## Footnotes

^1^ www.FDA.gov

## References

[1] Beghi E. The epidemiology of epilepsy. Neuroepidemiology. (2020) 54:185–91. doi: 10.1159/000503831, PMID: 31852003

[2] Kwan P Arzimanoglou A Berg AT Brodie MJ Allen Hauser W Mathern G. Definition of drug resistant epilepsy: consensus proposal by the ad hoc task force of the ILAE commission on therapeutic strategies. Epilepsia. (2010) 51:1069–77. doi: 10.1111/j.1528-1167.2009.02397.x, PMID: 19889013

[3] Gilioli I Vignoli A Visani E Casazza M Canafoglia L Chiesa V. Focal epilepsies in adult patients attending two epilepsy centers: classification of drug-resistance, assessment of risk factors, and usefulness of “new” antiepileptic drugs. Epilepsia. (2012) 53:733–40. doi: 10.1111/j.1528-1167.2012.03416.x, PMID: 22360822

[4] Mao J Takahashi K Cheng M Xu C Boca A Song Y. Real-world anti-seizure treatment and adverse events among individuals living with drug-resistant focal epilepsy in the United States. Epilepsia Open. (2024) 9:1311–20. doi: 10.1002/epi4.12967, PMID: 38818833 PMC11296083

[5] US Food and Drug Administration. *FDA approves new treatment for adults with partial-onset seizures*. Available online at: https://www.fda.gov/news-events/press-announcements/fda-approves-new-treatment-adults-partial-onset-seizures.

[6] European Medicines Agency. *Ontozry Cenobamate*. Available online at: https://www.ema.europa.eu/en/documents/scientific-conclusion/ontozry-h-c-psusa-00010921-202303-epar-scientific-conclusions-and-grounds-variation-terms-marketing-authorisation_en.pdf (Accessed 30 July 2024).

[7] Beltrán-Corbellini Á Romeral-Jiménez M Mayo P Sánchez-Miranda Román I Iruzubieta P Chico-García JL. Cenobamate in patients with highly refractory focal epilepsy: a retrospective real-world study. Seizure. (2023) 111:71–7. doi: 10.1016/j.seizure.2023.07.026, PMID: 37549616

[8] Makridis KL Kaindl AM. Real-world experience with cenobamate: a systematic review and meta-analysis. Seizure. (2023) 112:1–10. doi: 10.1016/j.seizure.2023.09.006, PMID: 37713961

[9] Nakamura M Cho JH Shin H Jang IS. Effects of cenobamate (YKP3089), a newly developed anti-epileptic drug, on voltage-gated sodium channels in rat hippocampal CA3 neurons. Eur J Pharmacol. (2019) 855:175–82. doi: 10.1016/j.ejphar.2019.05.007 31063770

[10] Sharma R Nakamura M Neupane C Jeon BH Shin H Melnick SM. Positive allosteric modulation of GABAA receptors by a novel antiepileptic drug cenobamate. Eur J Pharmacol. (2020) 879:173117. doi: 10.1016/j.ejphar.2020.173117, PMID: 32325146

[11] Roberti R De Caro C Iannone LF Zaccara G Lattanzi S Russo E. Pharmacology of cenobamate: mechanism of action, pharmacokinetics, drug-drug interactions and tolerability. CNS Drugs. (2021) 35:609–18. doi: 10.1007/s40263-021-00819-8, PMID: 33993416

[12] Krauss GL Klein P Brandt C Lee SK Milanov I Milovanovic M. Safety and efficacy of adjunctive cenobamate (YKP3089) in patients with uncontrolled focal seizures: a multicentre, double-blind, randomised, placebo-controlled, dose-response trial. Lancet Neurol. (2020) 19:38–48. doi: 10.1016/S1474-4422(19)30399-0, PMID: 31734103

[13] Hoeltzenbein M Slimi S Fietz AK Stegherr R Onken M Beyersmann J. Increasing use of newer antiseizure medication during pregnancy: an observational study with special focus on lacosamide. Seizure. (2023) 107:107–13. doi: 10.1016/j.seizure.2023.02.015, PMID: 37003184

[14] Rogawski MA Tofighy A White HS Matagne A Wolff C. Current understanding of the mechanism of action of the antiepileptic drug lacosamide. Epilepsy Res. (2015) 110:189–205. doi: 10.1016/j.eplepsyres.2014.11.021, PMID: 25616473 PMC13325623

[15] Wilson SM Khanna R. Specific binding of lacosamide to collapsin response mediator protein 2 (CRMP2) and direct impairment of its canonical function: implications for the therapeutic potential of lacosamide. Mol Neurobiol. (2015) 51:599–609. doi: 10.1007/s12035-014-8775-9, PMID: 24944082 PMC4272652

[16] Li J Sun M Wang X. The adverse-effect profile of lacosamide. Expert Opin Drug Saf. (2020) 19:131–8. doi: 10.1080/14740338.2020.1713089, PMID: 31914330

[17] Biton V Gil-Nagel A Isojarvi J Doty P Hebert D Fountain NB. Safety and tolerability of lacosamide as adjunctive therapy for adults with partial-onset seizures: analysis of data pooled from three randomized, double-blind, placebo-controlled clinical trials. Epilepsy Behav. (2015) 52:119–27. doi: 10.1016/j.yebeh.2015.09.006, PMID: 26414341

[18] Steinhoff BJ Ben-Menachem E Klein P Peltola J Schmitz B Thomas RH. Therapeutic strategies during cenobamate treatment initiation: Delphi panel recommendations. Ther Adv Neurol Disord. (2024) 17:733. doi: 10.1177/17562864241256733, PMID: 38883228 PMC11179501

[19] Huang L Guo T Zalkikar JN Tiwari RC. A review of statistical methods for safety surveillance. Ther Innov Regul Sci. (2014) 48:98–108. doi: 10.1177/2168479013514236, PMID: 30231423

[20] Fusaroli M Salvo F Begaud B AlShammari TM Bate A Battini V. The reporting of a disproportionality analysis for drug safety signal detection using individual case safety reports in PharmacoVigilance (READUS-PV): development and statement. Drug Saf. (2024) 47:575–84. doi: 10.1007/s40264-024-01421-9, PMID: 38713346 PMC11116242

[21] Steinhoff BJ Georgiou D Intravooth T. The cenobamate KORK study-A prospective monocenter observational study investigating cenobamate as an adjunctive therapy in refractory epilepsy, with comparisons to historical cohorts treated with add-on lacosamide, perampanel, and brivaracetam. Epilepsia Open. (2024) 9:1502–14. doi: 10.1002/epi4.12992, PMID: 38861254 PMC11296107

[22] Krauss GL Chung SS Ferrari L Stern S Rosenfeld WE. Cognitive and psychiatric adverse events during adjunctive cenobamate treatment in phase 2 and phase 3 clinical studies. Epilepsy Behav. (2024) 151:109605. doi: 10.1016/j.yebeh.2023.109605, PMID: 38184949

[23] Lauxmann S Heuer D Heckelmann J Fischer FP Schreiber M Schriewer E. Cenobamate: real-world data from a retrospective multicenter study. J Neurol. (2024) 271:6596–604. doi: 10.1007/s00415-024-12510-1, PMID: 38954033 PMC11447096

[24] Sperling MR Klein P Aboumatar S Gelfand M Halford JJ Krauss GL. Cenobamate (YKP3089) as adjunctive treatment for uncontrolled focal seizures in a large, phase 3, multicenter, open-label safety study. Epilepsia. (2020) 61:1099–108. doi: 10.1111/epi.16525, PMID: 32396252 PMC7317552

[25] Sperling MR Abou-Khalil B Aboumatar S Bhatia P Biton V Klein P. Efficacy of cenobamate for uncontrolled focal seizures: post hoc analysis of a phase 3, multicenter, open-label study. Epilepsia. (2021) 62:3005–15. doi: 10.1111/epi.17091, PMID: 34633084 PMC9293007

[26] Rosenfeld WE Abou-Khalil B Aboumatar S Bhatia P Biton V Krauss GL. Dose adjustments to concomitant antiseizure medications: post-hoc analysis of a phase 3, open-label study of cenobamate for treatment of unco2021ntrolled focal seizure. Neurology. (2021) 62:3016–28. doi: 10.1111/epi.17092, PMID: 34633074 PMC9292883

[27] Liu H Xu X. Influence of adjunctive lacosamide in patients with seizures: a systematic review and meta-analysis. Int J Neurosci. (2018) 128:670–6. doi: 10.1080/00207454.2017.1408619, PMID: 29172828

[28] Villanueva V Giráldez BG Toledo M De Haan GJ Cumbo E Gambardella A. Lacosamide monotherapy in clinical practice: a retrospective chart review. Acta Neurol Scand. (2018) 138:186–94. doi: 10.1111/ane.12920, PMID: 29542107 PMC6099342

[29] Makedonska I Ng YT Beller C Bozorg A Csikós J McClung C. Efficacy and tolerability of adjunctive lacosamide in patients aged <4 years with focal seizures. Ann Clin Transl Neurol. (2024) 11:768–79. doi: 10.1002/acn3.52004, PMID: 38375995 PMC10963285

[30] Zadeh WW Escartin A Byrnes W Tennigkeit F Borghs S Li T. Efficacy and safety of lacosamide as first add-on or later adjunctive treatment for uncontrolled partial-onset seizures: a multicentre open-label trial. Seizure. (2015) 31:72–9. doi: 10.1016/j.seizure.2015.07.001, PMID: 26362380

[31] Chen B Choi H Hirsch LJ Katz A Legge A Buchsbaum R. Psychiatric and behavioral side effects of antiepileptic drugs in adults with epilepsy. Epilepsy Behav. (2017) 76:24–31. doi: 10.1016/j.yebeh.2017.08.039, PMID: 28931473

[32] Hong Z Inoue Y Liao W Meng H Wang X Wang W. Efficacy and safety of adjunctive lacosamide for the treatment of partial-onset seizures in Chinese and Japanese adults: A randomized, double-blind, placebo-controlled study. Epilepsy Res. (2016) 127:267–75. doi: 10.1016/j.eplepsyres.2016.08.032, PMID: 27669155

[33] Li KY Huang LC Chang YP Yang YH. The effects of lacosamide on cognitive function and psychiatric profiles in patients with epilepsy. Epilepsy Behav. (2020) 113:107580. doi: 10.1016/j.yebeh.2020.107580, PMID: 33242771

[34] Nomoto M Konopaske GT Yamashita N Aoki R Jitsuki-Takahashi A Nakamura H. Clinical evidence that a dysregulated master neural network modulator may aid in diagnosing schizophrenia. Proc Natl Acad Sci U S A. (2021) 118:32118. doi: 10.1073/pnas.2100032118 PMC8346854 34330827

[35] Wang J Huang P Yu Q Lu J Liu P Yang Y. Epilepsy and long-term risk of arrhythmias. Eur Heart J. (2023) 44:3374–82. doi: 10.1093/eurheartj/ehad523, PMID: 37602368 PMC10499547

[36] Curia G Biagini G Perucca E Avoli M. Lacosamide: a new approach to target voltage-gated sodium currents in epileptic disorders. CNS Drugs. (2009) 23:555–68. doi: 10.2165/00023210-200923070-00002, PMID: 19552484 PMC4878900

[37] Rudd GD Haverkamp W Mason JW Wenger T Jay G Hebert D. Lacosamide cardiac safety: clinical trials in patients with partial-onset seizures. Acta Neurol Scand. (2015) 132:355–63. doi: 10.1111/ane.12414, PMID: 25933358

[38] Delaunois A Colomar A Depelchin BO Cornet M. Cardiac safety of lacosamide: the non-clinical perspective. Acta Neurol Scand. (2015) 132:337–45. doi: 10.1111/ane.12413, PMID: 25903789

[39] Tisdale JE Chung MK Campbell KB Hammadah M Joglar JA Leclerc J. Drug-induced arrhythmias: a scientific statement from the American Heart Association. Circulation. (2020) 142:e214–33. doi: 10.1161/CIR.0000000000000905, PMID: 32929996

[40] UCB Inc. *VIMPAT® (lacosamide) US Prescribing Information*. Available online at: https://www.ucb-usa.com/vimpat-prescribing-information.pdf (Accessed Jun 5, 2023).

[41] Wechsler RTS. Lacosamide In: Wyllie E, editor. Treatment of epilepsy. Philadelphia: Wolters Kluwer (2021). 641–5.

[42] Lachuer C Corny J Bézie Y Ferchichi S Durand-Gasselin B. Complete atrioventricular block in an elderly patient treated with low-dose lacosamide. Cardiovasc Toxicol. (2018) 18:579–82. doi: 10.1007/s12012-018-9467-x, PMID: 29948594

[43] Krause LU Brodowski KO Kellinghaus C. Atrioventricular block following lacosamide intoxication. Epilepsy Behav. (2011) 20:725–7. doi: 10.1016/j.yebeh.2011.02.006, PMID: 21411374

[44] Berei TJ Lillyblad MP Almquist AK. Lacosamide-induced recurrent ventricular tachycardia in the acute care setting. J Pharm Pract. (2018) 31:222–6. doi: 10.1177/0897190017700557, PMID: 28343443

[45] Yadav R Schrem E Yadav V Jayarangaiah A das S Theetha Kariyanna P. Lacosamide-related arrhythmias: a systematic analysis and review of the literature. Cureus. (2021) 13:e20736. doi: 10.7759/cureus.20736, PMID: 35111429 PMC8790938

[46] Shibata M Hoshino R Shimizu C Sato M Furuta N Ikeda Y. Lacosamide-induced sinus node dysfunction followed by severe agranulocytosis. BMC Neurol. (2021) 21:217. doi: 10.1186/s12883-021-02253-1, PMID: 34102997 PMC8185934

[47] Yang C Zhao W Chen H Yao Y Zhang J. Cardiac adverse events associated with lacosamide: a disproportionality analysis of the FAERS database. Sci Rep. (2024) 14:16202. doi: 10.1038/s41598-024-67209-0, PMID: 39003359 PMC11246456

[48] Ben-Menachem E Biton V Jatuzis D Abou-Khalil B Doty P Rudd GD. Efficacy and safety of oral lacosamide as adjunctive therapy in adults with partial-onset seizures. Epilepsia. (2007) 48:1308–17. doi: 10.1111/j.1528-1167.2007.01188.x, PMID: 17635557

[49] Vossler DG Wechsler RT Williams P Byrnes W Therriault S the ALEX‐MT study group. Long-term exposure and safety of lacosamide monotherapy for the treatment of partial-onset (focal) seizures: results from a multicenter, open-label trial. Epilepsia. (2016) 57:1625–33. doi: 10.1111/epi.13502, PMID: 27528101

[50] Kim HK Lee H Bae EK Kim DW. Cardiac effects of rapid intravenous loading of lacosamide in patients with epilepsy. Epilepsy Res. (2021) 176:106710. doi: 10.1016/j.eplepsyres.2021.106710, PMID: 34265537

[51] Lu YT Lin CH Ho CJ Hsu CW Tsai MH. Evaluation of cardiovascular concerns of intravenous lacosamide therapy in epilepsy patients. Front Neurol. (2022) 13:891368. doi: 10.3389/fneur.2022.891368, PMID: 35860491 PMC9289181

[52] Goodnough R Badea A Geier C Lynch KL LeSaint KT. Lacosamide induced brugada I morphology in the setting of septicemia: a case report. Medicine. (2021) 100:e25577. doi: 10.1097/MD.0000000000025577, PMID: 33950934 PMC8104292

[53] Lattanzi S Cagnetti C Foschi N Provinciali L Silvestrini M. Lacosamide during pregnancy and breastfeeding. Neurol Neurochir Pol. (2017) 51:266–9. doi: 10.1016/j.pjnns.2017.03.003, PMID: 28385340

[54] Kinney MO Smith PEM Craig JJ. Preventing teratogenicity in women with epilepsy. Semin Neurol. (2022) 42:679–92. doi: 10.1055/s-0042-1759579, PMID: 36513097

[55] Berman E Kohn E Berkovitch M Kovo M Eyal S. Lacosamide effects on placental carriers of essential compounds in comparison with valproate: studies in perfused human placentas. Epilepsia. (2022) 63:2949–57. doi: 10.1111/epi.17395, PMID: 36056753 PMC9826486

[56] Pack AM Oskoui M Williams Roberson S Donley DK French J Gerard EE. Teratogenesis, perinatal, and neurodevelopmental outcomes after in utero exposure to Antiseizure medication: practice guideline from the AAN, AES, and SMFM. Neurology. (2024) 102:e209279. doi: 10.1212/WNL.0000000000209279, PMID: 38748979 PMC11175651

[57] Smith MC Klein P Krauss GL Rashid S Seiden LG Stern JM. Dose adjustment of concomitant antiseizure medications during cenobamate treatment: expert opinion consensus recommendations. Neurol Ther. (2022) 11:1705–20. doi: 10.1007/s40120-022-00400-5 36057761 PMC9588096

[58] Osborn M Abou-Khalil B. The cenobamate-clobazam interaction- evidence of synergy in addition to pharmacokinetic interaction. Epilepsy Behav. (2023) 142:109156. doi: 10.1016/j.yebeh.2023.109156, PMID: 37037060

[59] Elakkary S Hagemann A Klimpel D Bien CG Brandt C. A retrospective non-interventional study evaluating the pharmacokinetic interactions between cenobamate and clobazam. Epilepsia. (2023) 64:e36–42. doi: 10.1111/epi.17515, PMID: 36661382

[60] Aboumatar S Ferrari L Stern S Wade CT Weingarten M Connor GS. Reductions in concomitant antiseizure medication drug load during adjunctive cenobamate therapy: post-hoc analysis of a subset of patients from a phase 3, multicenter, open-label study. Epilepsy Res. (2024) 200:107306. doi: 10.1016/j.eplepsyres.2024.107306, PMID: 38340681

[61] Becker DA Demko SA. Dose reduction and discontinuation of concomitant antiseizure medications after initiating cenobamate: A retrospective review. Epilepsy Res. (2023) 197:107242. doi: 10.1016/j.eplepsyres.2023.107242, PMID: 37871541

[62] Carreño M Gil-Nagel A Serratosa JM Toledo M Rodriguez-Uranga JJ Villanueva V. Spanish consensus on the management of concomitant antiseizure medications when using cenobamate in adults with drug-resistant focal seizures. Epilepsia Open. (2024) 9:1051–8. doi: 10.1002/epi4.12936, PMID: 38573131 PMC11145622

[63] Serrano-Castro PJ Rodríguez-Uranga JJ Cabezudo-García P García-Martín G Romero-Godoy J Estivill-Torrús G. Cenobamate and clobazam combination as personalized medicine in autoimmune-associated epilepsy with anti-GAD65 antibodies. Neurol Neuroimmunol Neuroinflamm. (2023) 10:e200151. doi: 10.1212/NXI.0000000000200151 37607753 PMC10443460

[64] Hung CC Chen CC Lin CJ Liou HH. Functional evaluation of polymorphisms in the human ABCB1 gene and the impact on clinical responses of antiepileptic drugs. Pharmacogenet Genomics. (2008) 18:390–402. doi: 10.1097/FPC.0b013e3282f85e36 18408562

[65] Zhao T Li HJ Feng J Zhang HL Ting-ting W Ma L. Impact of ABCB1 polymorphisms on Lacosamide serum concentrations in Uygur pediatric patients with epilepsy in China. Ther Drug Monit. (2022) 44:455–64. doi: 10.1097/FTD.0000000000000927, PMID: 34610620 PMC9083488

[66] Bjørke AB Nome CG Falk RS Gjerstad L Taubøll E Heuser K. Evaluation of long-term antiepileptic drug use in patients with temporal lobe epilepsy: assessment of risk factors for drug resistance and polypharmacy. Seizure. (2018) 61:63–70. doi: 10.1016/j.seizure.2018.07.011, PMID: 30099235

[67] Zierath D Mizuno S Barker-Haliski M. Frontline Sodium Channel-blocking Antiseizure medicine use promotes future onset of drug-resistant chronic seizures. Int J Mol Sci. (2023) 24:4848. doi: 10.3390/ijms24054848, PMID: 36902275 PMC10003379

[68] Pawluski JL Kuchenbuch M Hadjadj S Dieuset G Costet N Vercueil L. Long-term negative impact of an inappropriate first antiepileptic medication on the efficacy of a second antiepileptic medication in mice. Epilepsia. (2018) 59:e109–13. doi: 10.1111/epi.14454, PMID: 29901235

[69] Luoni C Bisulli F Canevini MP De Sarro G Fattore C Galimberti CA. Determinants of health-related quality of life in pharmacoresistant epilepsy: results from a large multicenter study of consecutively enrolled patients using validated quantitative assessments. Epilepsia. (2011) 52:2181–91. doi: 10.1111/j.1528-1167.2011.03325.x, PMID: 22136077

[70] Androsova G Krause R Borghei M Wassenaar M Auce P Avbersek A. Comparative effectiveness of antiepileptic drugs in patients with mesial temporal lobe epilepsy with hippocampal sclerosis. Epilepsia. (2017) 58:1734–41. doi: 10.1111/epi.13871, PMID: 28857179

[71] Perucca P Gilliam FG. Adverse effects of antiepileptic drugs. Lancet Neurol. (2012) 11:792–802. doi: 10.1016/S1474-4422(12)70153-9, PMID: 22832500

