# Supplementary material for: Real-world safety comparison between cenobamate and lacosamide: a pharmacovigilance study based on the FDA Adverse Event Reporting System
Source: Front Neurol. 2025 Sep 2;16:1625612. doi: 10.3389/fneur.2025.1625612 (PMC12439497; doi:10.3389/fneur.2025.1625612)
Supplement: Supplementary file 1 [file Supplementary_file_1.doc]

**Supplementary Data**

# Table S1. Ratio imbalance measurement algorithm.

| Item | Reports with the target AEs | All other AEs | Total |
| --- | --- | --- | --- |
| Reports with the target drug | a | b | a+b |
| All other drugs | c | d | c+d |
| Total | a+c | b+d | a+b+c+d |

# Table S2. Principle of dis-proportionality measure and standard of signal detection.

| Algorithms | Calculation formula | Criteria |
| --- | --- | --- |
| ROR | $ROR=\frac{a/c}{b/d}=\frac{ad}{bc}$ $95\%CI=e^{\ln(ROR)\pm1.96\sqrt{\frac{1}{a}+\frac{1}{b}+\frac{1}{c}+\frac{1}{d}}}$ | (1) a ≥ 6  (2) ROR ≥ 2  (3) 95%CI > 1 |
| PRR | $PRR=\frac{a/\left(a+b\right)}{c/\left(c+d\right)}=\frac{a\left(c+d\right)}{c\left(a+b\right)}$ $\chi^{2}=\frac{\left(\left\vert ad-bc\right\vert-\frac{n}{2}\right)^{2}n}{\left(a+b\right)\left(a+c\right)\left(c+d\right)\left(b+d\right)}$ n= a + b + c + d | (1) a ≥ 6  (2) PRR ≥ 2  (3)$\chi^{2}\geq4$ |
| BCPNN | $E(IC)=\log_{2}\frac{(C_{xy}+\gamma_{11})(C+\alpha)(C+\beta)}{(C+\gamma)(C_{x}+\alpha_{1})(C_{y}+\beta_{1})}$ | (1) a ≥ 6  (2) IC-2SD > 0 |
|  | $V(IC)=\frac{1}{(\ln2)^{2}}\left\{\left(\frac{C-C_{xy}+\gamma-\gamma_{11}}{(C_{xy}+\gamma_{11})(1+C+\gamma)}\right)+\left(\frac{C-C_{x}+\alpha-\alpha_{1}}{(C_{x}+\alpha_{1})(1+C+\alpha)}\right)+\left(\frac{C-C_{y}+\beta-\beta_{1}}{(C_{y}+\beta_{1})(1+C+\beta)}\right)\right\}$ $\gamma=\gamma_{11}\frac{(C+\alpha)(C+\beta)}{(C_{x}+\alpha_{1})(C_{y}+\beta_{1})}$ $IC-2SD=E(IC)-2\sqrt{V(IC)}$ $\alpha_{1}=\beta_{1}=1;\alpha=\beta=2;\gamma_{11}=1;$ $C=a+b+c+d;C_{x}=a+b;C_{y}=a+c;C_{xy}=a$ | |

Abbreviations: ROR: Reporting odds ratio; PRR：Proportional Reporting Ratio ; BCPNN: Bayesian confidence propagation neural network; CI: Confidence Interval; IC: Information Component

# Table S3. Adverse event signals of Lacosamide and Cenobamate in different organ system categories.

| **SOCs / PTs** | **Lacosamide** | | | | | **Cenobamate** | | | | |
| --- | --- | --- | --- | --- | --- | --- | --- | --- | --- | --- |
|  | **N** | **PRR** | **Chi_ squared** | **ROR(CI025)** | **IC(IC-2SD)** | **N** | **PRR** | **Chi_ squared** | **ROR(CI025)** | **IC(IC-2SD)** |
| **Cardiac Disorders** | | | | | | | | | | |
| aortic valve incompetence | 6 | 2.51 | 5.44 | 2.51(1.13~5.59) | 1.33(0.23) | / | / | / | / | / |
| arrhythmia | 71 | 2.35 | 55.07 | 2.35(1.86~2.97) | 1.23(0.89) | / | / | / | / | / |
| atrial flutter | 37 | 7.07 | 191.94 | 7.08(5.12~9.78) | 2.82(2.35) | / | / | / | / | / |
| atrioventricular block | 93 | 19.2 | 1580.72 | 19.25(15.68~23.63) | 4.24(3.94) | / | / | / | / | / |
| atrioventricular block complete | 100 | 24.23 | 2185.12 | 24.29(19.92~29.61) | 4.57(4.28) | / | / | / | / | / |
| atrioventricular block first degree | 36 | 13.14 | 399.56 | 13.15(9.47~18.26) | 3.7(3.22) | / | / | / | / | / |
| atrioventricular block second degree | 52 | 27.73 | 1310.93 | 27.77(21.09~36.56) | 4.76(4.36) | / | / | / | / | / |
| bradycardia | 220 | 6.39 | 996.87 | 6.42(5.62~7.34) | 2.67(2.48) | / | / | / | / | / |
| bradycardia neonatal | 14 | 17.55 | 215.5 | 17.56(10.36~29.76) | 4.11(3.36) | / | / | / | / | / |
| bundle branch block left | 24 | 9.18 | 173.69 | 9.19(6.15~13.73) | 3.19(2.61) | / | / | / | / | / |
| bundle branch block right | 18 | 6.42 | 81.93 | 6.42(4.04~10.21) | 2.68(2.01) | / | / | / | / | / |
| cardiac arrest | 118 | 2.21 | 77.79 | 2.21(1.84~2.65) | 1.14(0.88) | / | / | / | / | / |
| conduction disorder | 11 | 10.71 | 96.04 | 10.71(5.92~19.4) | 3.41(2.57) | / | / | / | / | / |
| defect conduction intraventricular | 6 | 26.64 | 144.95 | 26.64(11.87~59.81) | 4.71(3.6) | / | / | / | / | / |
| pulseless electrical activity | 7 | 2.47 | 6.11 | 2.47(1.18~5.18) | 1.3(0.28) | / | / | / | / | / |
| sinus arrest | 23 | 24.97 | 518.88 | 24.98(16.53~37.76) | 4.61(4.02) | / | / | / | / | / |
| sinus bradycardia | 48 | 7.94 | 289.48 | 7.95(5.99~10.56) | 2.98(2.57) | / | / | / | / | / |
| sinus node dysfunction | 34 | 27.7 | 856.15 | 27.73(19.73~38.95) | 4.76(4.27) | / | / | / | / | / |
| supraventricular extrasystoles | 8 | 3.97 | 17.73 | 3.97(1.98~7.95) | 1.99(1.02) | / | / | / | / | / |
| ventricular arrhythmia | 6 | 2.55 | 5.64 | 2.55(1.14~5.68) | 1.35(0.26) | / | / | / | / | / |
| ventricular fibrillation | 19 | 2.83 | 22.38 | 2.83(1.8~4.44) | 1.5(0.85) | / | / | / | / | / |
| ventricular tachycardia | 41 | 4.07 | 94.68 | 4.07(3~5.54) | 2.02(1.58) | / | / | / | / | / |
| electrocardiogram pr prolongation | 21 | 48.6 | 942.38 | 48.63(31.44~75.21) | 5.55(4.92) | / | / | / | / | / |
| electrocardiogram qrs complex prolonged | 17 | 5.5 | 62.29 | 5.5(3.42~8.86) | 2.45(1.77) | / | / | / | / | / |
| electrocardiogram qt prolonged | 54 | 2.26 | 37.99 | 2.26(1.73~2.96) | 1.18(0.79) | / | / | / | / | / |
| **Congenital, Familial and Genetic Disorders** | | | | | | | | | | |
| atrial septal defect | 33 | 5.41 | 118.19 | 5.42(3.85~7.62) | 2.43(1.93) | / | / | / | / | / |
| brugada syndrome | 9 | 13.81 | 105.77 | 13.81(7.16~26.64) | 3.77(2.85) | / | / | / | / | / |
| cardiac septal defect | 10 | 20.28 | 180.4 | 20.29(10.86~37.9) | 4.32(3.44) | / | / | / | / | / |
| coarctation of the aorta | 20 | 29.28 | 533.83 | 29.3(18.8~45.65) | 4.84(4.2) | / | / | / | / | / |
| congenital hydronephrosis | 11 | 22.76 | 224.71 | 22.76(12.54~41.33) | 4.48(3.64) | / | / | / | / | / |
| cryptorchism | 8 | 10.79 | 70.44 | 10.79(5.38~21.64) | 3.42(2.45) | / | / | / | / | / |
| cytogenetic abnormality | 7 | 8.4 | 45.3 | 8.4(3.99~17.66) | 3.06(2.04) | / | / | / | / | / |
| dysmorphism | 6 | 3.41 | 10.18 | 3.41(1.53~7.6) | 1.77(0.67) | / | / | / | / | / |
| foetal malformation | 17 | 24.66 | 378.42 | 24.67(15.26~39.87) | 4.6(3.91) | / | / | / | / | / |
| hepatic arteriovenous malformation | 7 | 545.77 | 2647.91 | 545.86(224.55~1326.92) | 8.57(7.39) | / | / | / | / | / |
| multiple congenital abnormalities | 11 | 11.28 | 102.18 | 11.29(6.23~20.44) | 3.48(2.65) | / | / | / | / | / |
| patent ductus arteriosus | 9 | 3.16 | 13.22 | 3.16(1.64~6.07) | 1.66(0.74) | / | / | / | / | / |
| polydactyly | 7 | 8.84 | 48.32 | 8.84(4.2~18.59) | 3.13(2.11) | / | / | / | / | / |
| spina bifida | 7 | 4.54 | 19.27 | 4.54(2.16~9.54) | 2.18(1.16) | / | / | / | / | / |
| trisomy 18 | 6 | 24.78 | 134.28 | 24.79(11.05~55.62) | 4.6(3.5) | / | / | / | / | / |
| **Ear and Labyrinth Disorders** | | | | | | | | | | |
| hyperacusis | 6 | 2.48 | 5.27 | 2.48(1.11~5.52) | 1.31(0.21) | / | / | / | / | / |
| vertigo | 89 | 2.18 | 57.15 | 2.19(1.78~2.69) | 1.13(0.82) | 31 | 2.66 | 32.16 | 2.67(1.87~3.79) | 1.41(0.9) |
| **Eye Disorders** | | | | | | | | | | |
| blepharospasm | 12 | 3.4 | 20.26 | 3.4(1.93~5.99) | 1.76(0.96) | / | / | / | / | / |
| diplopia | 174 | 10.46 | 1477.28 | 10.5(9.04~12.2) | 3.38(3.16) | 83 | 17.39 | 1277.97 | 17.51(14.11~21.74) | 4.12(3.8) |
| ocular discomfort | / | / | / | / | / | 6 | 3.11 | 8.59 | 3.11(1.4~6.93) | 1.64(0.55) |
| vision blurred | / | / | / | / | / | 83 | 3.27 | 131.12 | 3.29(2.65~4.08) | 1.71(1.39) |
| visual impairment | / | / | / | / | / | 37 | 1.53 | 6.77 | 1.53(1.11~2.11) | 0.61(0.14) |
| **Gastrointestinal Disorders** | | | | | | | | | | |
| dysphagia | 30 | 1.69 | 8.42 | 1.69(1.18~2.42) | 0.75(0.24) | / | / | / | / | / |
| hypoaesthesia oral | 26 | 2.6 | 25.48 | 2.6(1.77~3.82) | 1.37(0.82) | / | / | / | / | / |
| **General Disorders and Administration Site Conditions** | | | | | | | | | | |
| adverse drug reaction | / | / | / | / | / | 27 | 1.6 | 6.15 | 1.61(1.1~2.34) | 0.68(0.14) |
| crying | 50 | 2.05 | 26.96 | 2.05(1.56~2.71) | 1.04(0.63) | 22 | 3.16 | 32.45 | 3.16(2.08~4.81) | 1.66(1.06) |
| drug interaction | / | / | / | / | / | 91 | 3.08 | 127.83 | 3.09(2.52~3.8) | 1.62(1.32) |
| drug intolerance | / | / | / | / | / | 51 | 2.71 | 54.88 | 2.71(2.06~3.57) | 1.44(1.03) |
| fatigue | / | / | / | / | / | 432 | 2.87 | 531.85 | 2.94(2.67~3.24) | 1.52(1.38) |
| feeling abnormal | / | / | / | / | / | 172 | 3.65 | 331.89 | 3.69(3.17~4.29) | 1.87(1.65) |
| feeling drunk | 27 | 5.5 | 98.94 | 5.5(3.77~8.03) | 2.45(1.91) | 35 | 24.98 | 801.33 | 25.06(17.96~34.95) | 4.63(4.15) |
| gait disturbance | / | / | / | / | / | 142 | 3.69 | 279.36 | 3.73(3.16~4.4) | 1.88(1.64) |
| gait inability | / | / | / | / | / | 32 | 5.25 | 110.02 | 5.26(3.72~7.45) | 2.39(1.89) |
| multiple-drug resistance | 177 | 95.75 | 15413.47 | 96.17(82.51~112.09) | 6.48(6.25) | / | / | / | / | / |
| screaming | 15 | 3.64 | 28.58 | 3.64(2.19~6.04) | 1.86(1.14) | 7 | 5.93 | 28.66 | 5.93(2.83~12.45) | 2.57(1.55) |
| sudden death | 13 | 2.01 | 6.61 | 2.01(1.17~3.47) | 1.01(0.24) | / | / | / | / | / |
| thirst | / | / | / | / | / | 10 | 2.98 | 13.14 | 2.98(1.6~5.54) | 1.57(0.7) |
| **Infections and Infestations** | | | | | | | | | | |
| herpes simplex | 8 | 2.47 | 7 | 2.47(1.24~4.95) | 1.3(0.34) | / | / | / | / | / |
| **Injury, Poisoning and Procedural Complications** | | | | | | | | | | |
| contusion | / | / | / | / | / | 28 | 1.51 | 4.9 | 1.52(1.05~2.2) | 0.6(0.06) |
| fall | 584 | 2.62 | 585.52 | 2.64(2.43~2.87) | 1.39(1.27) | 213 | 3.34 | 350.3 | 3.38(2.95~3.87) | 1.74(1.54) |
| limb injury | / | / | / | / | / | 10 | 1.93 | 4.45 | 1.93(1.04~3.58) | 0.94(0.07) |
| maternal drugs affecting foetus | 13 | 2.45 | 11.17 | 2.45(1.42~4.23) | 1.29(0.52) | / | / | / | / | / |
| **Investigations** | | | | | | | | | | |
| ammonia increased | 8 | 2.41 | 6.58 | 2.41(1.2~4.82) | 1.27(0.3) | / | / | / | / | / |
| anticoagulation drug level abnormal | 8 | 72.84 | 535.59 | 72.86(35.71~148.64) | 6.11(5.11) | / | / | / | / | / |
| anticonvulsant drug level above therapeutic | 9 | 23.59 | 191.05 | 23.59(12.2~45.62) | 4.53(3.61) | / | / | / | / | / |
| anticonvulsant drug level decreased | 14 | 16.86 | 206.06 | 16.86(9.95~28.58) | 4.06(3.31) | / | / | / | / | / |
| anticonvulsant drug level increased | 15 | 15.08 | 194.82 | 15.08(9.06~25.1) | 3.9(3.17) | / | / | / | / | / |
| blood lactic acid increased | 9 | 2.78 | 10.22 | 2.78(1.44~5.34) | 1.47(0.56) | / | / | / | / | / |
| blood sodium decreased | 35 | 3.02 | 47.29 | 3.02(2.17~4.22) | 1.59(1.11) | 7 | 2.11 | 4.1 | 2.11(1.01~4.43) | 1.08(0.06) |
| drug level decreased | 18 | 2.69 | 19.08 | 2.69(1.69~4.27) | 1.43(0.76) | / | / | / | / | / |
| drug level increased | 21 | 2 | 10.45 | 2(1.3~3.07) | 1(0.38) | 7 | 2.33 | 5.3 | 2.33(1.11~4.89) | 1.22(0.2) |
| electrocardiogram abnormal | 12 | 2.39 | 9.66 | 2.39(1.36~4.21) | 1.25(0.45) | / | / | / | / | / |
| gamma-glutamyltransferase increased | 27 | 2.06 | 14.61 | 2.06(1.41~3) | 1.04(0.49) | / | / | / | / | / |
| **Metabolism and Nutrition Disorders** | | | | | | | | | | |
| cell death | 6 | 4.4 | 15.72 | 4.4(1.98~9.82) | 2.13(1.04) | / | / | / | / | / |
| decreased appetite | / | / | / | / | / | 83 | 1.88 | 34.05 | 1.88(1.52~2.34) | 0.91(0.59) |
| hyperammonaemia | 14 | 4.36 | 36.17 | 4.36(2.58~7.38) | 2.12(1.37) | / | / | / | / | / |
| hypernatraemia | 8 | 2.49 | 7.12 | 2.49(1.24~4.98) | 1.31(0.35) | / | / | / | / | / |
| marasmus | 7 | 7.33 | 38.01 | 7.33(3.49~15.4) | 2.87(1.84) | / | / | / | / | / |
| polydipsia | 6 | 2.48 | 5.29 | 2.48(1.11~5.53) | 1.31(0.22) | / | / | / | / | / |
| vitamin b12 deficiency | 6 | 2.31 | 4.44 | 2.31(1.04~5.14) | 1.21(0.11) | / | / | / | / | / |
| **Musculoskeletal and Connective Tissue Disorders** | | | | | | | | | | |
| limb discomfort | / | / | / | / | / | 12 | 1.92 | 5.33 | 1.93(1.09~3.39) | 0.94(0.14) |
| **Nervous System Disorders** | | | | | | | | | | |
| eye movement disorder | 16 | 3.53 | 28.99 | 3.53(2.16~5.77) | 1.82(1.12) | 13 | 10.05 | 105.67 | 10.06(5.83~17.34) | 3.33(2.55) |
| altered state of consciousness | 58 | 4 | 130.11 | 4(3.09~5.18) | 2(1.62) | / | / | / | / | / |
| amnesia | 196 | 4.63 | 557.19 | 4.65(4.04~5.35) | 2.21(2) | 41 | 3.38 | 68.88 | 3.39(2.5~4.61) | 1.76(1.31) |
| aphasia | 86 | 4.24 | 212.49 | 4.25(3.44~5.25) | 2.08(1.77) | 30 | 5.17 | 100.81 | 5.18(3.62~7.41) | 2.37(1.85) |
| apraxia | 6 | 6.34 | 26.84 | 6.34(2.84~14.14) | 2.66(1.56) | / | / | / | / | / |
| ataxia | 62 | 7.53 | 349.07 | 7.54(5.87~9.68) | 2.91(2.54) | 15 | 6.35 | 67.51 | 6.36(3.83~10.55) | 2.66(1.94) |
| balance disorder | 213 | 3.61 | 401.1 | 3.62(3.17~4.15) | 1.85(1.65) | 140 | 8.3 | 897.83 | 8.39(7.1~9.91) | 3.05(2.81) |
| bradykinesia | 9 | 2.4 | 7.36 | 2.4(1.25~4.62) | 1.26(0.35) | / | / | / | / | / |
| brain fog | / | / | / | / | / | 9 | 6.14 | 38.68 | 6.14(3.19~11.82) | 2.62(1.7) |
| cerebral disorder | 18 | 3.71 | 35.48 | 3.71(2.33~5.89) | 1.89(1.22) | / | / | / | / | / |
| clumsiness | 7 | 3.76 | 14.14 | 3.76(1.79~7.9) | 1.91(0.89) | 6 | 11.28 | 56.07 | 11.29(5.06~25.15) | 3.49(2.4) |
| cognitive disorder | 97 | 3.08 | 136.07 | 3.09(2.53~3.77) | 1.62(1.33) | 23 | 2.55 | 21.72 | 2.56(1.7~3.85) | 1.35(0.76) |
| coma | 70 | 2.39 | 56.25 | 2.39(1.89~3.02) | 1.25(0.91) | / | / | / | / | / |
| coordination abnormal | 30 | 3.49 | 53.17 | 3.49(2.44~5) | 1.8(1.28) | 21 | 8.55 | 139.68 | 8.56(5.58~13.14) | 3.09(2.48) |
| dementia | 52 | 2.9 | 64.56 | 2.9(2.21~3.81) | 1.53(1.14) | / | / | / | / | / |
| depressed level of consciousness | 56 | 2.38 | 44.68 | 2.38(1.83~3.09) | 1.25(0.87) | / | / | / | / | / |
| disturbance in attention | 96 | 2.65 | 98.32 | 2.65(2.17~3.24) | 1.4(1.11) | 47 | 4.53 | 129.41 | 4.55(3.41~6.06) | 2.18(1.76) |
| dizziness | 694 | 2.12 | 412.65 | 2.14(1.98~2.31) | 1.08(0.97) | 344 | 3.67 | 674.26 | 3.76(3.37~4.18) | 1.88(1.72) |
| drop attacks | 14 | 35.5 | 456.37 | 35.51(20.87~60.41) | 5.11(4.35) | / | / | / | / | / |
| drug withdrawal convulsions | 23 | 18.81 | 382.2 | 18.82(12.47~28.42) | 4.21(3.62) | / | / | / | / | / |
| dysarthria | 63 | 2.59 | 61.34 | 2.59(2.02~3.32) | 1.37(1.01) | 61 | 8.77 | 419.62 | 8.82(6.85~11.34) | 3.13(2.76) |
| dysgraphia | / | / | / | / | / | 8 | 6.12 | 34.24 | 6.13(3.06~12.26) | 2.61(1.65) |
| dyskinesia | 58 | 2.16 | 36.06 | 2.16(1.67~2.8) | 1.11(0.73) | 18 | 2.34 | 13.86 | 2.35(1.48~3.72) | 1.23(0.57) |
| dyslexia | 6 | 7.88 | 35.81 | 7.88(3.53~17.58) | 2.97(1.88) | / | / | / | / | / |
| dysstasia | / | / | / | / | / | 29 | 5.08 | 94.87 | 5.09(3.53~7.32) | 2.34(1.81) |
| febrile convulsion | 8 | 10.86 | 70.99 | 10.86(5.41~21.79) | 3.43(2.46) | / | / | / | / | / |
| head discomfort | / | / | / | / | / | 11 | 3.22 | 16.84 | 3.22(1.78~5.82) | 1.69(0.85) |
| headache | / | / | / | / | / | 230 | 1.91 | 100.27 | 1.93(1.69~2.19) | 0.93(0.74) |
| hypersomnia | 49 | 2.57 | 47 | 2.57(1.94~3.41) | 1.36(0.95) | 88 | 16.2 | 1250.72 | 16.31(13.22~20.13) | 4.01(3.71) |
| lethargy | / | / | / | / | / | 58 | 5.44 | 210.21 | 5.46(4.22~7.07) | 2.44(2.07) |
| loss of consciousness | 208 | 2.57 | 199.29 | 2.58(2.25~2.95) | 1.36(1.16) | 49 | 2.12 | 28.86 | 2.12(1.6~2.81) | 1.08(0.67) |
| memory impairment | 289 | 3.04 | 395.77 | 3.06(2.72~3.43) | 1.6(1.43) | 120 | 4.41 | 317.26 | 4.45(3.72~5.33) | 2.14(1.88) |
| migraine | / | / | / | / | / | 38 | 2.12 | 22.63 | 2.13(1.55~2.93) | 1.09(0.62) |
| motor dysfunction | 8 | 4.19 | 19.4 | 4.19(2.09~8.38) | 2.07(1.1) | / | / | / | / | / |
| nystagmus | 27 | 8.05 | 165.6 | 8.05(5.52~11.76) | 3(2.45) | 7 | 7.27 | 37.79 | 7.27(3.46~15.27) | 2.86(1.84) |
| psychomotor skills impaired | 7 | 3.86 | 14.81 | 3.86(1.84~8.12) | 1.95(0.92) | / | / | / | / | / |
| sedation | 50 | 3.13 | 72.34 | 3.13(2.37~4.14) | 1.64(1.24) | 29 | 6.35 | 130.56 | 6.36(4.42~9.16) | 2.66(2.14) |
| slow speech | / | / | / | / | / | 11 | 23.41 | 234.74 | 23.43(12.95~42.39) | 4.54(3.71) |
| somnolence | 425 | 3.24 | 657.37 | 3.26(2.96~3.59) | 1.69(1.55) | 406 | 10.82 | 3622.68 | 11.18(10.13~12.35) | 3.43(3.29) |
| speech disorder | 99 | 2.85 | 118.66 | 2.85(2.34~3.48) | 1.51(1.22) | 36 | 3.62 | 68.33 | 3.63(2.62~5.04) | 1.86(1.38) |
| syncope | 157 | 2.38 | 125.98 | 2.39(2.04~2.79) | 1.25(1.02) | / | / | / | / | / |
| tongue biting | 7 | 5.52 | 25.79 | 5.52(2.63~11.6) | 2.46(1.44) | / | / | / | / | / |
| tremor | 236 | 2.16 | 147.12 | 2.17(1.91~2.46) | 1.11(0.92) | 79 | 2.53 | 73.12 | 2.54(2.03~3.17) | 1.34(1.01) |
| bradyphrenia | 12 | 2.59 | 11.65 | 2.59(1.47~4.56) | 1.37(0.57) | 6 | 4.52 | 16.44 | 4.52(2.03~10.08) | 2.18(1.08) |
| **Pregnancy, Puerperium and Perinatal Conditions** | | | | | | | | | | |
| abortion | 6 | 3.01 | 8.06 | 3.01(1.35~6.72) | 1.59(0.5) | / | / | / | / | / |
| abortion spontaneous | 131 | 4.94 | 410.24 | 4.95(4.17~5.88) | 2.3(2.05) | / | / | / | / | / |
| gestational diabetes | 8 | 2.54 | 7.45 | 2.54(1.27~5.08) | 1.34(0.38) | / | / | / | / | / |
| hydrops foetalis | 8 | 19.49 | 138.19 | 19.5(9.7~39.2) | 4.26(3.29) | / | / | / | / | / |
| low birth weight baby | 15 | 2.64 | 15.29 | 2.64(1.59~4.39) | 1.4(0.68) | / | / | / | / | / |
| premature baby | 58 | 2.78 | 66.14 | 2.79(2.15~3.6) | 1.47(1.1) | / | / | / | / | / |
| premature delivery | 49 | 4.22 | 120.14 | 4.23(3.19~5.6) | 2.07(1.67) | / | / | / | / | / |
| small for dates baby | 9 | 2.71 | 9.65 | 2.71(1.41~5.2) | 1.43(0.52) | / | / | / | / | / |
| stillbirth | 19 | 5.78 | 74.71 | 5.78(3.68~9.07) | 2.52(1.88) | / | / | / | / | / |
| **Psychiatric Disorders** | | | | | | | | | | |
| mental impairment | / | / | / | / | / | 18 | 3.86 | 38.2 | 3.87(2.44~6.14) | 1.95(1.29) |
| abnormal behaviour | 107 | 4.17 | 256.66 | 4.17(3.45~5.05) | 2.05(1.78) | 39 | 5.3 | 136.11 | 5.32(3.88~7.28) | 2.41(1.95) |
| abnormal dreams | / | / | / | / | / | 11 | 2.07 | 6.08 | 2.07(1.15~3.74) | 1.05(0.22) |
| acute psychosis | 15 | 9.38 | 111.46 | 9.38(5.65~15.59) | 3.22(2.5) | / | / | / | / | / |
| affective disorder | 23 | 4.42 | 60.77 | 4.43(2.94~6.67) | 2.14(1.55) | 6 | 4.03 | 13.66 | 4.03(1.81~8.98) | 2.01(0.92) |
| aggression | 152 | 4.72 | 444.57 | 4.74(4.04~5.56) | 2.24(2) | 31 | 3.36 | 51.42 | 3.37(2.37~4.79) | 1.75(1.24) |
| agitation | 127 | 2.68 | 133.85 | 2.69(2.26~3.2) | 1.42(1.17) | 36 | 2.66 | 37.24 | 2.66(1.92~3.69) | 1.41(0.93) |
| anger | 89 | 3.91 | 192.66 | 3.92(3.18~4.83) | 1.97(1.66) | 34 | 5.23 | 116.1 | 5.24(3.74~7.34) | 2.38(1.9) |
| anxiety | / | / | / | / | / | 74 | 1.34 | 6.37 | 1.34(1.07~1.69) | 0.42(0.09) |
| apathy | 26 | 2.73 | 28.44 | 2.73(1.86~4.01) | 1.45(0.89) | 14 | 5.14 | 46.64 | 5.14(3.05~8.69) | 2.36(1.61) |
| behaviour disorder | 34 | 10.48 | 289 | 10.48(7.48~14.69) | 3.38(2.89) | 11 | 11.8 | 108.42 | 11.81(6.53~21.34) | 3.56(2.72) |
| catatonia | 7 | 2.11 | 4.08 | 2.11(1.01~4.43) | 1.08(0.05) | / | / | / | / | / |
| communication disorder | / | / | / | / | / | 6 | 8.57 | 40.07 | 8.58(3.85~19.11) | 3.1(2.01) |
| confusional state | / | / | / | / | / | 77 | 2.55 | 72.95 | 2.56(2.05~3.21) | 1.35(1.02) |
| delirium | 67 | 3.05 | 91.91 | 3.05(2.4~3.88) | 1.6(1.25) | / | / | / | / | / |
| delusion | 21 | 2.13 | 12.55 | 2.13(1.39~3.27) | 1.09(0.47) | / | / | / | / | / |
| depression | / | / | / | / | / | 64 | 1.47 | 9.6 | 1.47(1.15~1.88) | 0.55(0.2) |
| depression suicidal | 7 | 2.92 | 8.84 | 2.92(1.39~6.14) | 1.55(0.52) | / | / | / | / | / |
| disorientation | 59 | 2.33 | 44.7 | 2.33(1.81~3.01) | 1.22(0.85) | 18 | 2.48 | 15.97 | 2.49(1.57~3.95) | 1.31(0.65) |
| emotional disorder | / | / | / | / | / | 20 | 3.36 | 33.22 | 3.37(2.17~5.22) | 1.75(1.12) |
| euphoric mood | 6 | 3.07 | 8.37 | 3.07(1.38~6.84) | 1.62(0.53) | / | / | / | / | / |
| fear | / | / | / | / | / | 11 | 2.03 | 5.78 | 2.03(1.13~3.68) | 1.02(0.19) |
| hallucination | 97 | 2.02 | 49.76 | 2.02(1.65~2.47) | 1.01(0.72) | / | / | / | / | / |
| hallucination, auditory | / | / | / | / | / | 7 | 2.39 | 5.66 | 2.39(1.14~5.02) | 1.26(0.24) |
| hallucination, visual | 30 | 2.32 | 22.51 | 2.32(1.62~3.32) | 1.21(0.69) | / | / | / | / | / |
| homicidal ideation | 12 | 4.76 | 35.47 | 4.76(2.7~8.39) | 2.25(1.44) | / | / | / | / | / |
| impulse-control disorder | 6 | 4.5 | 16.28 | 4.5(2.02~10.03) | 2.17(1.07) | / | / | / | / | / |
| impulsive behaviour | 7 | 2.94 | 8.93 | 2.94(1.4~6.17) | 1.55(0.53) | / | / | / | / | / |
| inappropriate affect | 6 | 5.58 | 22.46 | 5.58(2.5~12.45) | 2.48(1.38) | / | / | / | / | / |
| insomnia | / | / | / | / | / | 95 | 1.87 | 38.69 | 1.88(1.53~2.3) | 0.9(0.61) |
| intentional self-injury | / | / | / | / | / | 8 | 2.02 | 4.14 | 2.02(1.01~4.05) | 1.02(0.05) |
| irritability | 126 | 3.14 | 182.93 | 3.14(2.64~3.74) | 1.65(1.39) | 36 | 3.13 | 52.2 | 3.14(2.26~4.35) | 1.65(1.17) |
| logorrhoea | 10 | 4.3 | 25.27 | 4.3(2.31~8.01) | 2.1(1.23) | / | / | / | / | / |
| mood altered | / | / | / | / | / | 25 | 4.85 | 76.25 | 4.85(3.28~7.19) | 2.28(1.71) |
| mood swings | 55 | 2.56 | 52.37 | 2.57(1.97~3.34) | 1.36(0.97) | 21 | 3.42 | 36 | 3.43(2.23~5.26) | 1.77(1.16) |
| nervousness | / | / | / | / | / | 22 | 2.19 | 14.25 | 2.19(1.44~3.33) | 1.13(0.53) |
| panic attack | 49 | 2.05 | 26.48 | 2.06(1.55~2.72) | 1.04(0.63) | 13 | 1.91 | 5.6 | 1.91(1.11~3.29) | 0.93(0.16) |
| paranoia | 34 | 3.09 | 48.05 | 3.09(2.21~4.33) | 1.63(1.14) | 7 | 2.23 | 4.72 | 2.23(1.06~4.67) | 1.15(0.13) |
| persecutory delusion | 10 | 6.45 | 45.85 | 6.45(3.47~12.02) | 2.68(1.81) | / | / | / | / | / |
| personality change | 26 | 4.19 | 62.95 | 4.19(2.85~6.16) | 2.06(1.51) | 13 | 7.32 | 70.86 | 7.33(4.25~12.63) | 2.87(2.1) |
| psychiatric symptom | 14 | 2.83 | 16.54 | 2.83(1.68~4.78) | 1.5(0.75) | / | / | / | / | / |
| psychomotor retardation | 11 | 6.15 | 47.24 | 6.15(3.4~11.13) | 2.62(1.78) | / | / | / | / | / |
| psychotic disorder | 71 | 3.81 | 146.65 | 3.81(3.02~4.81) | 1.93(1.59) | / | / | / | / | / |
| self-injurious ideation | 6 | 2.27 | 4.23 | 2.27(1.02~5.05) | 1.18(0.09) | / | / | / | / | / |
| staring | 14 | 9.27 | 102.53 | 9.27(5.48~15.69) | 3.2(2.45) | 7 | 16.17 | 99.23 | 16.18(7.7~33.98) | 4.01(2.99) |
| stress | / | / | / | / | / | 37 | 2.66 | 38.45 | 2.67(1.93~3.69) | 1.41(0.94) |
| suicidal ideation | 141 | 2.36 | 110.72 | 2.37(2.01~2.79) | 1.24(1) | 54 | 3.16 | 79.96 | 3.17(2.43~4.15) | 1.66(1.27) |
| dysphemia | 11 | 3.77 | 22.37 | 3.78(2.09~6.82) | 1.91(1.08) | 11 | 13.22 | 123.84 | 13.23(7.32~23.91) | 3.72(2.89) |
| thinking abnormal | / | / | / | / | / | 11 | 3.1 | 15.69 | 3.11(1.72~5.61) | 1.63(0.8) |
| **Reproductive System and Breast Disorders** | | | | | | | | | | |
| erectile dysfunction | / | / | / | / | / | 13 | 2.86 | 15.7 | 2.86(1.66~4.93) | 1.51(0.74) |
| **Respiratory, Thoracic and Mediastinal Disorders** | | | | | | | | | | |
| aspiration | 20 | 3.01 | 26.71 | 3.01(1.94~4.66) | 1.59(0.95) | / | / | / | / | / |
| hiccups | / | / | / | / | / | 14 | 9.71 | 109.12 | 9.72(5.75~16.42) | 3.28(2.53) |
| **Skin and Subcutaneous Tissue Disorders** | | | | | | | | | | |
| drug eruption | 34 | 3.04 | 46.58 | 3.05(2.18~4.27) | 1.6(1.12) | / | / | / | / | / |
| erythema multiforme | 13 | 2.25 | 9.01 | 2.25(1.31~3.88) | 1.17(0.4) | / | / | / | / | / |
| lichenoid keratosis | 7 | 5.22 | 23.8 | 5.22(2.49~10.97) | 2.38(1.36) | / | / | / | / | / |
| rash pruritic | / | / | / | / | / | 18 | 1.82 | 6.6 | 1.82(1.14~2.89) | 0.86(0.2) |
| skin warm | 8 | 2.06 | 4.33 | 2.06(1.03~4.11) | 1.04(0.08) | / | / | / | / | / |
| stevens-johnson syndrome | 33 | 2.53 | 30.58 | 2.53(1.8~3.57) | 1.34(0.84) | / | / | / | / | / |
| toxic epidermal necrolysis | 20 | 2.13 | 11.94 | 2.13(1.37~3.3) | 1.09(0.46) | / | / | / | / | / |
| **Social Circumstances** | | | | | | | | | | |
| impaired driving ability | 15 | 2.11 | 8.69 | 2.11(1.27~3.5) | 1.07(0.35) | 6 | 2.95 | 7.71 | 2.95(1.32~6.56) | 1.56(0.47) |
| impaired quality of life | / | / | / | / | / | 11 | 4.09 | 25.67 | 4.09(2.27~7.4) | 2.03(1.2) |
| impaired work ability | / | / | / | / | / | 18 | 4.35 | 46.42 | 4.36(2.74~6.92) | 2.12(1.46) |
| loss of personal independence in daily activities | / | / | / | / | / | 17 | 1.91 | 7.32 | 1.91(1.18~3.07) | 0.93(0.25) |
| **Vascular Disorders** | | | | | | | | | | |
| systolic hypertension | 7 | 24.67 | 155.87 | 24.67(11.68~52.13) | 4.6(3.57) | / | / | / | / | / |

Abbreviations: PTs, Preferred Terms; SOCs, System Organ Classes; N is the number of reported adverse events; IC (IC-2SD), information component (lower end of the 95% confidence interval); /, IC-2SD value of the adverse event is less than 0.
